# Robustness of RNA sequencing on older formalin-fixed paraffin-embedded tissue from high-grade ovarian serous adenocarcinomas

**DOI:** 10.1371/journal.pone.0216050

**Published:** 2019-05-06

**Authors:** Yongmei Zhao, Monika Mehta, Ashley Walton, Keyur Talsania, Yelena Levin, Jyoti Shetty, Elizabeth M. Gillanders, Bao Tran, Danielle Mercatante Carrick

**Affiliations:** 1 NCI CCR Sequencing Facility, Frederick National Laboratory for Cancer Research, Frederick, MD, United States of America; 2 Advanced Biomedical and Computational Sciences, Frederick National Laboratory for Cancer Research, Frederick, MD, United States of America; 3 Division of Cancer Control and Population Sciences (DCCPS), National Cancer Institute, Rockville, MD, United States of America; Centro Nacional de Investigaciones Oncologicas, SPAIN

## Abstract

Formalin-fixed paraffin-embedded (FFPE) tissues are among the most widely available clinical specimens. Their potential utility as a source of RNA for transcriptome studies would greatly enhance population-based cancer studies. Although preliminary studies suggest FFPE tissue may be used for RNA sequencing, the effect of storage time on these specimens needs to be determined. We conducted this study to determine whether RNA in archived FFPE high-grade ovarian serous adenocarcinomas from Surveillance, Epidemiology and End Results (SEER) registries was present in sufficient quantity and quality for RNA-Seq analysis. FFPE tissues, stored from 7 to 32 years, were obtained from three SEER sites. RNA was extracted, quantified, quality assessed, and subjected to RNA-Seq (a whole transcriptome sequencing technology). FFPE specimens stored for longer periods of time had poorer RNA sample quality as indicated by negative correlations between specimen storage time and fragment distribution values (DV). In addition, sample contamination was a common issue among the RNA, with 41 of 67 samples having 5% to 48% bacterial contamination. However, regardless of specimen storage time and bacterial contamination, 60% of the samples yielded data that enabled gene expression quantification, identifying more than 10,000 genes, with the correlations among most biological replicates above 0.7. This study demonstrates that FFPE high-grade ovarian serous adenocarcinomas specimens stored in repositories for up to 32 years and under varying storage conditions are a promising source of RNA for RNA-Seq. We also describe certain caveats to be considered when designing RNA-Seq studies using archived FFPE tissues.

## Introduction

Advances in molecular technologies have enabled more comprehensive molecular characterization of pathology tissues beyond what can be achieved using morphological or clinical factors. The wide application of next-generation sequencing of both DNA and RNA has revolutionized cancer research. Comprehensive analyses of different cancer types in The Cancer Genome Atlas (TCGA) have revealed many genomic changes that can be used as markers of tumor subtypes and may represent new therapeutic targets. For example, gene expression-based subclassification of glioblastoma into subtypes appears to be more predictive of treatment response than traditional methods of using clinical or morphological factors [1–3].

Fresh frozen tissue is the desired sample type for all molecular analyses. However, tissue that is normally left after surgery and diagnosis is typically formalin fixed and paraffin embedded (FFPE) due to the cost effectiveness of storing tissues preserved in this manner versus freezing tissues at ultra-low temperatures [4]. Therefore, FFPE tissues are generally the most commonly available samples for epidemiology studies. DNA from archival FFPE tissue samples can be used for next generation sequencing (NGS) methods such as whole exome sequencing [5, 6].

RNA from FFPE tissues may be more prone to being chemically modified, cross-linked and degraded over time due to fixation and archiving methods than DNA, and therefore, whole transcriptome analyses may be more difficult than DNA analysis [7]. Despite those potential difficulties, several studies suggest RNA from FFPE tissues may be used for RNA-Seq, a whole transcriptome sequencing method that utilizes NGS [6–10]. If the RNA is sufficiently preserved, RNA-Seq using FFPE-derived RNA has been shown to yield transcriptome data that are comparable to that obtained using RNA from frozen tissue for colorectal, prostate, and bladder cancers [6], in addition to glioblastoma [8], renal [9], and lung tissues [10]. Hedegard et. al. [6] showed that RNA-seq can be used with RNA from FFPE tissue blocks stored for up to 20 years; however, many stored tissue specimens may be considerably older. As studies such as ones by Vukmirovic et al [10] and Esteve-Codina et al [8] demonstrate, while FFPE tissue can be used for RNA-Seq, not all of the specimens yield usable data for reasons that are not yet understood. Here, we expand on published FFPE for RNAseq studies by increasing the number of archived FFPE tissues examined and including a wider range of specimen storage times. Our study also includes suggestions for identifying RNA from FFPE tissue that might be predicted to yield unusable RNA-Seq data.

The Surveillance, Epidemiology, and End Results (SEER) cancer registries cover approximately 28% of the United States population, providing high quality demographic, clinical, pathologic, and survival data. In three of the SEER registries, annotated FFPE tumor tissue specimens were made available for research through established Residual Tissue Repositories (RTR) [11]. The FFPE tissues from SEER were procured from clinical pathology labs and were processed and stored in non-uniform manners. These tissues are therefore reflective of the types of tissue material that could be used for translational clinical research.

We performed this study to determine whether it is feasible to perform RNA-Seq assays using FFPE-derived RNA obtained from 60 high-grade ovarian serous adenocarcinomas (plus 7 replicates) from SEER Residual Tissue Repositories (RTR) that were stored up to 32 years, and to develop some criteria to help understand which FFPE tissues may not yield useful RNA-Seq data.

## Materials and methods

### Human subjects protections

This study was approved by institutional review boards (IRB) at participating cancer registries (University of Southern California Health Sciences Campus, The University of Iowa and the University of Hawaii) and at the National Cancer Institute (NCI). The study was determined to be exempt from IRB review under 45 CFR 46.101(b)(4) for the use of anonymized and coded or coded but unlinked tissue blocks from the SEER registries. No contact with subjects was made for this study.

### Subject/specimen selection

The same FFPE tissue sections used in our previous analysis of SEER FFPE tissues for whole exome sequencing were used in this study [5] and from the same cases as were used for proteomics analysis of FFPE tissues [12]. As specimens were retrospectively collected by the RTRs from multiple medical facilities and pathology labs within each of the three catchment areas, fixation times/conditions (such as type of formalin used) and storage conditions (such as temperature, controlled humidity, etc.) were unknown. Tissues were from high-grade serous ovarian adenocarcinomas (ICD-O-3 Topography code: C56.9; Morphology codes: 8441/3, 8460/3, 8461/3) and storage times ranged from 7 to 32 years; storage times were calculated from the date when the cancer was diagnosed to the date when RNA was extracted (storage times were rounded up to the next year). For seven cases, two different sections, or replicates, were prepared and sent to the lab for analysis (Table 1). The lab performed each study procedure on these sections in a blinded fashion. The sections were then used to assess consistency of results.

**Table 1:** FFPE specimens selected for analysis.

	Specimen Storage Time					
	7–12 years	13–22 years	23–32 years	Unknown Age	Replicates*	Total
SEER site 1	8	9	3	0	4	24
SEER site 2	4	11	5	0	3	23
SEER site 3	1	11	7	1	0	20
Total	13	31	15	1	7	67

*For seven cases two separate specimens were prepared and sent to the laboratory.

Each SEER registry conducted a pathology review of lead and trail sections flanking the 5 sections from each tissue block to determine whether tissue was consistent with the selection criteria (high-grade serous ovarian adenocarcinoma, ≥ 50% of cells with nuclei consistent with malignant cells, and ≤ 50% of cells were necrotic); approximately 30 cases from each registry were reviewed to ultimately select 20 cases that met study criteria. For each case identified as meeting the study criteria, five 10-micron sections were placed in a sterile tube. Additional details regarding the pathology review are described previously [5].

### DNA/RNA extraction and RNA quantity and quality assessment

The FFPE tissues were deparaffinized using xylene and ethanol according to the procedures detailed by Qiagen for the AllPrep DNA/RNA FFPE Kit. RNA was eluted in 60 microliters EB buffer (Qiagen). Qiagen All Prep FFPE kit was used to purify DNA and RNA from each specimen; the entire tissue section sent was used for nucleic acid purification (i.e. no macrodissection was performed). RNA quality was checked by running on the Agilent Bioanalyzer (RNA Nano chip) and RNA Integrity Number (RIN) values were obtained. The RNA quality was also assessed using the RT^2^ RNA QC PCR Array from Qiagen that tests for the presence of transcripts of two housekeeping genes, *ACTB* and *HPRT1*, in addition to testing for inhibitors of Reverse Transcription and PCR amplification reactions, and the presence of genomic DNA contamination.

### Library preparation

NGS libraries were prepared using the NEBNext Ultra II RNA Library Prep Kit for Illumina. The input amounts of RNA used for library preparation ranged from 21 ng to 1000 ng. The input amount of RNA (Table 2) was determined by the amount of sample available. Wherever >2000 ng RNA was available (36 samples), the maximum amount per reaction (1000 ng) was used as the input. For samples where the total amount available was between 2000 ng and 500 ng, half of the RNA was used for library preparation, and the other half was saved for repeating the library prep, if needed. For samples with less than 500 ng available, the entire RNA was used for library preparation. The volume used for the library prep ranged from 0.6 ul to 8.6 ul, depending on the RNA concentration. None of the samples were concentrated using SpeedVac or any other means.

**Table 2:** All Samples QC Results (sorted by specimen storage time).

Sample Name	Input for lib prep (ng)	RIN	DV_200_	DV_100_	DV_50_	Lib size (bp)	Lib yield (ng)	Lib Molarity (nM)	Specimen storage time (Years)	*Resequenced?	% contamination	Gene Expression Cluster (Fig 8)	Gene Count
			(%)	(%)	(%)								
SEER_020	672.3	2.3	30	75	98	413	34.8	10.3	7	N	22.61	B	14216
SEER_064	1000	2.3	42	73	90	478	78	18.6	7	Y	55.47	C	13844
SEER_041	1000	2.5	11	41	98	428	8.28	2.4	9	N	4.69	D	4541
SEER_042	1000	2.2	39	81	99	334	5467.95	190	9	Y	0.38	B	4850
SEER_043	1000	2.4	14	50	97	421	19.8	5.9	9	N	13.31	D	6429
SEER_044	232.4	2.7	NA	NA	NA	393	7.26	2.5	9	N	4.25	D	16093
SEER_045	1000	2.4	16	55	98	368	12.5	4.6	9	Y	9.08	D	14766
SEER_065	1000	2.3	21	64	98	456	3.14	0.84	9	Y	23.83	D	60
SEER_060	747	2.6	6	30	94	445	4.55	1.2	10	N	24.75	B	27053
SEER_061	1000	2.2	50	86	100	331	246	91	10	Y	0.48	B	5169
SEER_016	597.8	2.5	17	60	98	375	985.95	363.2	12	N	39.06		15313
SEER_017	1000	1	NA	NA	NA	365	3.69	1.15	12	N	2.04	D	24435
SEER_019	1000	2.4	21	64	98	393	207.9	66	12	N	12.3	B	22417
SEER_021	1000	2.4	18	67	99	337	417.45	154.9	12	N	1.22	B	9993
SEER_052	391.05	2.4	11	43	96	405	8.55	2.6	12	N	3.95	D	15398
SEER_013	1000	2.1	35	81	99	312	286.8	105	13	Y	0.51	B	17729
SEER_014	568	2.5	17	66	99	355	10.47	3.33	13	N	3.16	B	21893
SEER_015	1024	2.4	13	56	98	364	456.75	159.4	13	N	31.93		5688
SEER_018	1000	2.1	36	82	100	290	216.9	83	13	Y	0.08	C	21580
SEER_022	1000	2.6	4	42	98	372	197.55	43.4	13	N	2.06	C	20620
SEER_023	1000	2.4	5	34	97	371	389.1	145.1	13	N	33.26		22385
SEER_051	1000	2.4	15	56	98	405	9.77	3	13	N	8.39	D	9062
SEER_012	907.5	NA	10	65	99	329	28.13	9.46	14	Y	1.22	B	18627
SEER_050	1000	2.4	13	59	98	376	21	6.6	14	Y	7.22	D	16584
SEER_066	1000	2.4	12	56	99	426	86.1	26	14	N	21.89	D	17
SEER_029	237.6	2.5	12	35	93	426	24.45	7	15	Y	27.38	D	31105
SEER_049	2000	1.7	NA	NA	NA	446	6.56	1.5	15	N	7.09	D	3380
SEER_010	1000	1.1	NA	NA	NA	315	44.21	15.23	16	Y	0.58	B	17485
SEER_011	682.5	2.2	NA	NA	NA	411	1.11	0.3	16	N	3.76		22
SEER_027	830.7	2.5	9	51	98	385	84.75	27.7	16	N	9.36	B	9056
SEER_009	369.75	NA	NA	NA	NA	353	8.58	2.65	17	Y	5.11	B	17832
SEER_048	1000	2.4	15	55	97	399	13.62	4.2	17	N	10.27	C	5981
SEER_007	1000	1	NA	NA	NA	408	5.04	1.43	18	Y	1.71	C	42
SEER_008	4731	1.1	NA	NA	NA	307	211.35	82.7	18	Y	0.08	D	8177
SEER_025	576.2	2.5	11	50	97	408	50.25	14.7	18	N	24.04	B	20527
SEER_026	1000	2.5	8	48	97	409	59.7	17.6	18	N	22.36	B	8392
SEER_046	1000	2.4	12	48	98	404	17.4	5.4	18	Y	9.89	D	4703
SEER_047	1000	2.3	22	67	99	387	23.25	7.3	18	N	10.9	D	5145
SEER_063	1000	2.3	27	77	99	356	124.2	41	18	N	0.49	B	10920
SEER_006	392.2	NA	NA	NA	NA	450	2.49	0.622	19	Y	12.02	B	12652
SEER_024	926.55	2.5	12	57	99	360	207.75	67.9	20	N	1.04	C	22926
SEER_028	697.2	2.5	9	46	96	433	38.4	10.9	20	N	23.14	B	10202
SEER_036	336.7	2.8	18	59	98	410	8.79	2.7	21	N	9.18	D	4115
SEER_057	1000	2.2	26	68	98	571	5.81	1.4	21	N	24.29	D	14391
SEER_058	1000	2.4	12	51	97	479	4.89	1.3	21	Y	29.53	D	27840
SEER_059	1000	2.4	12	53	97	520	8.3	2	21	N	18.6	C	23689
SEER_067	1000	2.3	24	71	99	462	84.9	24	21	N	40.39	C	24
SEER_034	1000	2.2	47	85	98	352	49.05	16	22	N	5	C	9
SEER_035	716.8	2.3	33	79	89	294	218.25	76.6	22	N	1.99	B	4000
SEER_056	21.6	NA	4	24	92	444	28.2	7.3	22	N	35.6	B	8966
SEER_037	356.25	2.6	10	47	95	406	6.63	2.04	23	N	4.38	D	8645
SEER_038	1000	2.5	13	55	97	448	6.68	1.9	23	N	8.5	D	12
SEER_039	1000	2.5	14	57	98	408	11.75	3.6	23	N	5.36	D	16370
SEER_040	93.6	1.5	7	38	76	479	15.9	4	23	Y	24.54	D	9782
SEER_055	94.6	2.3	1	11	85	450	30	7.6	23	N	35.71	B	2448
SEER_005	1000	1.1	NA	NA	NA	382	5.07	1.53	24	Y	2.69	C	8963
SEER_053	315.25	2.6	9	36	89	547	11.3	2.5	24	N	18.53	C	14759
SEER_054	1000	2.5	7	46	93	548	8.33	1.8	24	Y	41.29	B	4020
SEER_062	1000	2.3	22	74	99	344	366	126	24	N	0.41	B	4152
SEER_004	1000	2.6	NA	NA	NA	427	4.22	1.13	25	N	8.43	D	25
SEER_030	413.4	2.5	16	54	97	433	27.3	7.7	25	Y	30.66	D	10431
SEER_031	537.95	2.5	11	51	97	428	36.45	10.3	25	N	44.1	C	13883
SEER_032	256.5	2.5	13	49	96	377	84.3	26.9	25	N	6.66	C	28255
SEER_003	1000	1.1	NA	NA	NA	381	4.77	1.44	27	N	1.95	D	24
SEER_002	799.5	2.1	NA	NA	NA	402	3.2	0.882	29	N	6.47	D	17
SEER_001	696.8	NA	NA	NA	NA	424	3.8	0.983	31	Y	5.31	B	60
SEER_033	440.7	NA	21	63	98	417	67.35	19.6	no info	Y	39.79	D	3210

*Resequenced = one of the 25 samples that were resequenced due to low sequencing yields. NA = Values were not able to be obtained and were not included in statistical analyses.

Libraries were prepared per manufacturer recommendations. Briefly, the ribosomal RNA (rRNA) was depleted by hybridization to rRNA probes, followed by RNase H digestion of the hybridized RNA. RNase H is a better method than RiboZero for depleting ribosomal RNA when low quality RNAs, such as from FFPE tissues, are anticipated [7]. Excess probes were removed by DNase I digestion, followed by RNA purification using Agencourt RNAClean XP beads. Because the samples had small sized fragments to begin with, no fragmentation was done during the library prep. First strand cDNA was synthesized by ProtoScript II Reverse Transcriptase using random priming at 42°C, for 30–45 mins. Second strand cDNA synthesis by nick translation was enabled by RNase H by creating nicks and gaps in the RNA strand. Ends of the cDNA were then repaired to make them blunt and phosphorylated, followed by dA-tailing and adaptor ligation. Sample indexes were added during the PCR enrichment step. 15–19 cycles of amplification were used. PCR reactions were purified using Agencourt SPRIselect beads (0.9X). Samples with additional peaks at ~80bp (primers) or 128bp (adaptor-dimers) in the bioanalyzer traces underwent additional clean ups to eliminate the residual primers or adaptor dimers. Libraries were QC’ed by running on the Bioanalyzer. The average library fragment size ranged from 290bp to 571bp (median: 405bp), and library yields ranged from 1 ng to 5 ug, with the median yield of 23.2 ng.

### RNA sequencing

The libraries were sequenced on Illumina NextSeq 500 using NextSeq High Output v2 kit. Three samples were sequenced using the pair-end run mode with run setup as 2x76 bps. The rest of the 64 samples were sequenced on NextSeq 500 using the 1x151 bps single-read run with 10 samples pooled per run; this was done as recommended by Illumina tech support when the initial pair-end 2x76bps runs did not work for Read 2. In addition, 25 samples (25 out of 67; 37%) with low sequencing yield were re-sequenced on NextSeq 500 using the 1x151 bps runs to get additional sequencing depth.

### Contamination assessment

Bacterial contamination of the RNA was determined for all samples using the FastScreen (https://www.bioinformatics.babraham.ac.uk/projects/fastq_screen) software tool. The mapping step in the software filtered out the bacteria reads.

### Bioinformatics and statistical analysis

Illumina’s HiSeq Real Time Analysis software (RTA) was used for processing images files, and the Illumina bcl2fastq_v2.17 was used for demultiplexing. The quality of reads was investigated using FastQC (https://www.bioinformatics.babraham.ac.uk/projects/fastqc) to detect any abnormalities in the raw sequencing reads. FastqScreen was also used to detect any reads originating from other species indicating bacterial contamination. The sequencing adapters and low quality bases were trimmed using Trimmomatic [13] software (version 0.30). The trimmed reads were aligned to human genome reference sequence GRCh Build 38 using STAR aligner version 2.5.1. The Gencode annotation version 24 was used to guide the spliced transcript alignment. Picard’s MarkDuplicates (https://broadinstitute.github.io/picard/command-line-overview.html#MarkDuplicates) was used to evaluate the library complexity by determining the amount of unique or non-duplicated reads in the samples. Picard’s CollectRnaSeqMetrics program (https://broadinstitute.github.io/picard/command-line-overview.html) was used to collect mapping percentages on coding, intronic, intergenic and UTR regions as well as gene body coverage. In addition, RSeQC [14] version 2.3.5 was used to determine read pair inner distance, read GC content, junction saturation, and library strand. Additional QC statistics were generated by using multiQC [15] software package. RSEM [16] version 1.2.22 was used to get raw count as well as normalized read count on genes. Genes expressed below a noised threshold (TPM<1 or raw count <5) were filtered. Evidence of batch effects was analyzed using a Principal Components Analysis (PCA) analysis using an R script. Correlation analysis was carried out using the Pearson correlation between different metrics. R packages were used to generate sample correlation plots. See S1 Table for software pipeline and parameters.

The biological replicates were handled as independent samples since the bacterial contamination, percent of mapped reads, and percent of mRNA reads were very different for replicates, as well as the libraries were prepared independently for each replicate sample. The correlation between the replicates was calculated.

## Results

### RNA quality assessment

**Fig 1:**
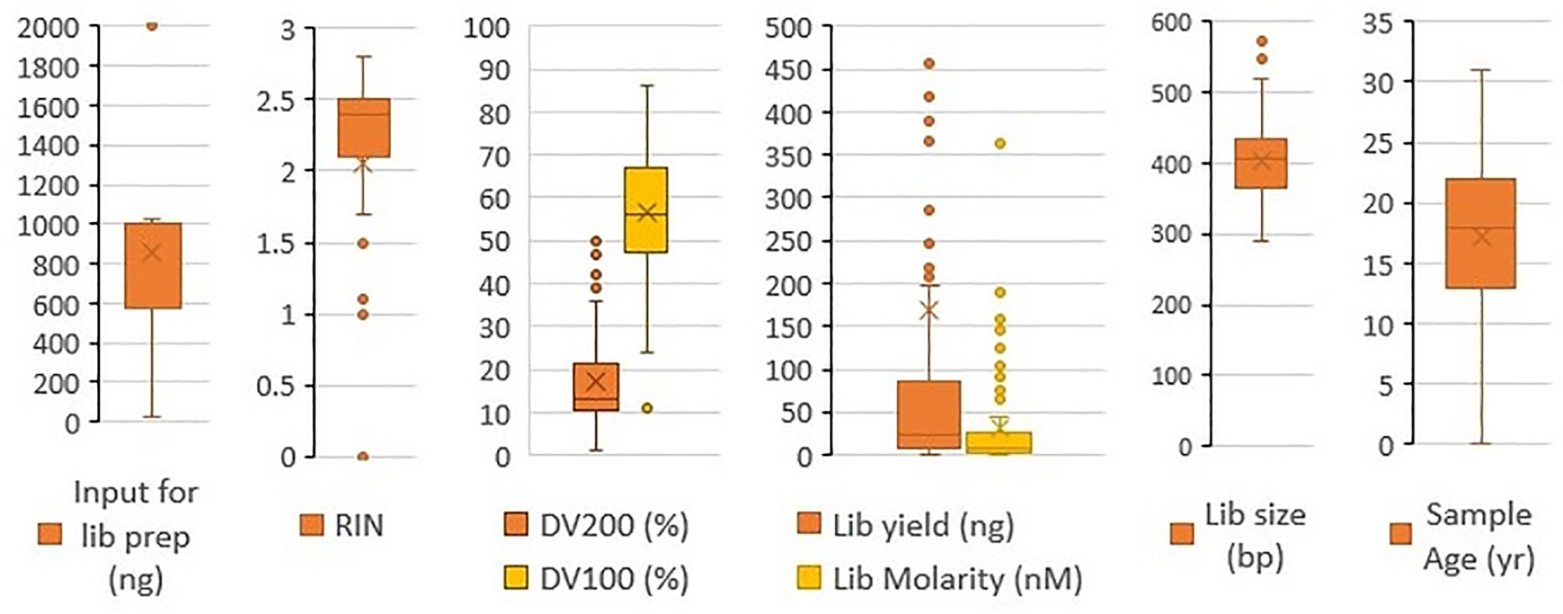
Sample and library QC box plots. The plots the median, mean and standard deviation of sample storage age, RIN, fragment distribution values (DV) (DV_200_ and DV_100_), input amount for library, library yield, library molarity, and library size distributions. For each box and whisker plot, the middle line of the box represents the median, and the ‘x’ represents the mean.The top and bottom lines represent 75th and 25th percentile, respectively. The whiskers extend to the data point 1.5 times the interquartile range above the 3rd quartile or below the first quartile. Values outside this range are represented by dots. Extreme outliers in the plots for input for library preparation (4731 ng and 2000 ng) and library yield (5467.95 ng and 985.95 ng) have been omitted for clarity of visualization of the distributions. Values that could not be obtained are designated as N/A in Table 2 and were not included in Fig 1.

RNA extracted from the 67 FFPE samples obtained from the SEER repositories was assessed for quality before preparing NGS libraries (Fig 1). The RIN values of the RNA samples could be calculated for 61 of the 67 samples, and were between 1 and 2.8, with a median value of 2.4 (Fig 1 and Table 2). To better assess the degradation of the samples, fragment distribution values (DV) were calculated to estimate the percentage of fragments longer than 200 nt (DV_200_), 100 nt (DV_100_), and 50 nt (DV_50_). The DV_200_ (percentage of RNA fragments > 200 nt in size) was between 1% and 50%, with a median value of 13%. The DV_100_ (percentage of RNA fragments > 100 nt in size) was between 11% and 86%, with a median value of 56%. DV_50_ (percentage of RNA fragments > 50 nt in size) was found to be between 76% and 100%, with a median value of 98%; see Table 2. DV estimates could not be made for 14 samples; this could have been due to the fragment distribution in the sample not allowing the software to accurately identify the markers that are used to estimate the fragment size.

**Fig 2:**
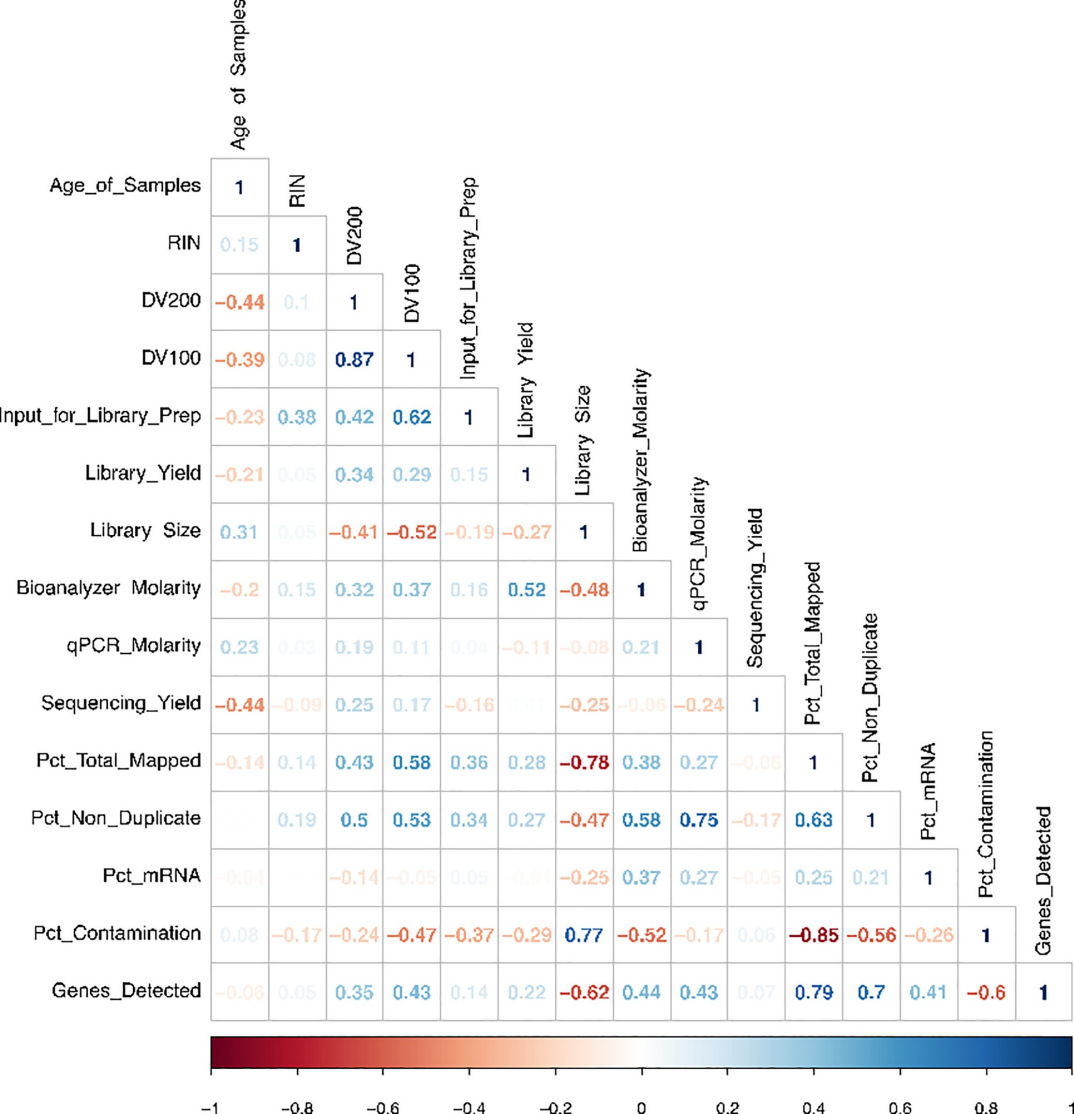
Correlations analysis based on QC results. Correlations analysis based on QC results and sample metadata. Pearson correlation coefficients were calculated for a matrix of the various parameters examined in this study; the correlation matrix using the R package corrplot. The parameters included in the matrix were: age of samples in years (Age of Samples), RIN, DV_200_, DV_100_, library input of starting material (Input_for_Library_Prep), Bioanalyzer measurement of library yield in ng (Library_Yield), library size in base pairs (Library Size), molarity of the libraries in nM (Bioanalyzer Molarity), qPCR molarity, sequencing yield, total percentage of mapped reads to human (Pct_Total_Mapped), total percentage of non-duplicate mapped reads (Pct_Non_Duplicate), percent mRNA bases mapped to human (Pct_mRNA), total percentage of mapped reads to bacterial and mouse genomes (Pct_Contamination), and total number of genes which have more than 5 read mapped (Genes_Detected). Positive correlations are displayed in blue and negative correlations in red color. Color intensity is proportional to the correlation coefficients. In the bottom of the correlogram, the legend color shows the correlation coefficients and the corresponding colors. Values between 0.7 and 1.0 (-0.7 and -1.0) indicate a strong positive (negative) linear relationship. Samples with NA values were excluded.

Shorter fragments are a common characteristic of more degraded RNA. When comparing specimen storage times with DV_100_ and DV_200_ values, there was a negative correlation between the storage time and DV values (-0.44 for DV_200_ and -0.39 for DV_100_) indicating that longer storage time is associated with more degraded RNA (Fig 2); specimens stored for 7 to 12 years had an average DV_200_ of 20 and DV_100_ of 53, those stored 13 to 22 years yielded an average DV_200_ of 14 and DV_100_ of 46, and those stored 23–32 years yielded an average DV_200_ of 8 and DV_100_ of 34. In addition, there was a weak negative correlation between the age and library yield (-0.21); specimens stored for 7 to 12 years yielded an average library of 500 ng, stored 13 to 22 years yielded an average library of 87ng, and those stored 23–32 years yielded an average library of 41ng. This was expected since degraded samples have less amplifiable RNA and therefore, have lower library yields compared to non-degraded RNAs when using the same PCR conditions. We did not find the RIN values to be a useful measure of quality for the highly degraded FFPE RNA samples (Fig 2). DV_200_, though a better metric than RIN, was also not the best QC metric for these samples, since highly degraded RNA fragment sizes were less than 200 nt; therefore, the DV_200_ values were very low. Thus, we decided to calculate DV_100_ and DV_50_. DV_100_ gave the most useful measure of fragment distribution, and thus, sample quality for these samples, as is evident from the correlation plot in Fig 2.

**Fig 3:**
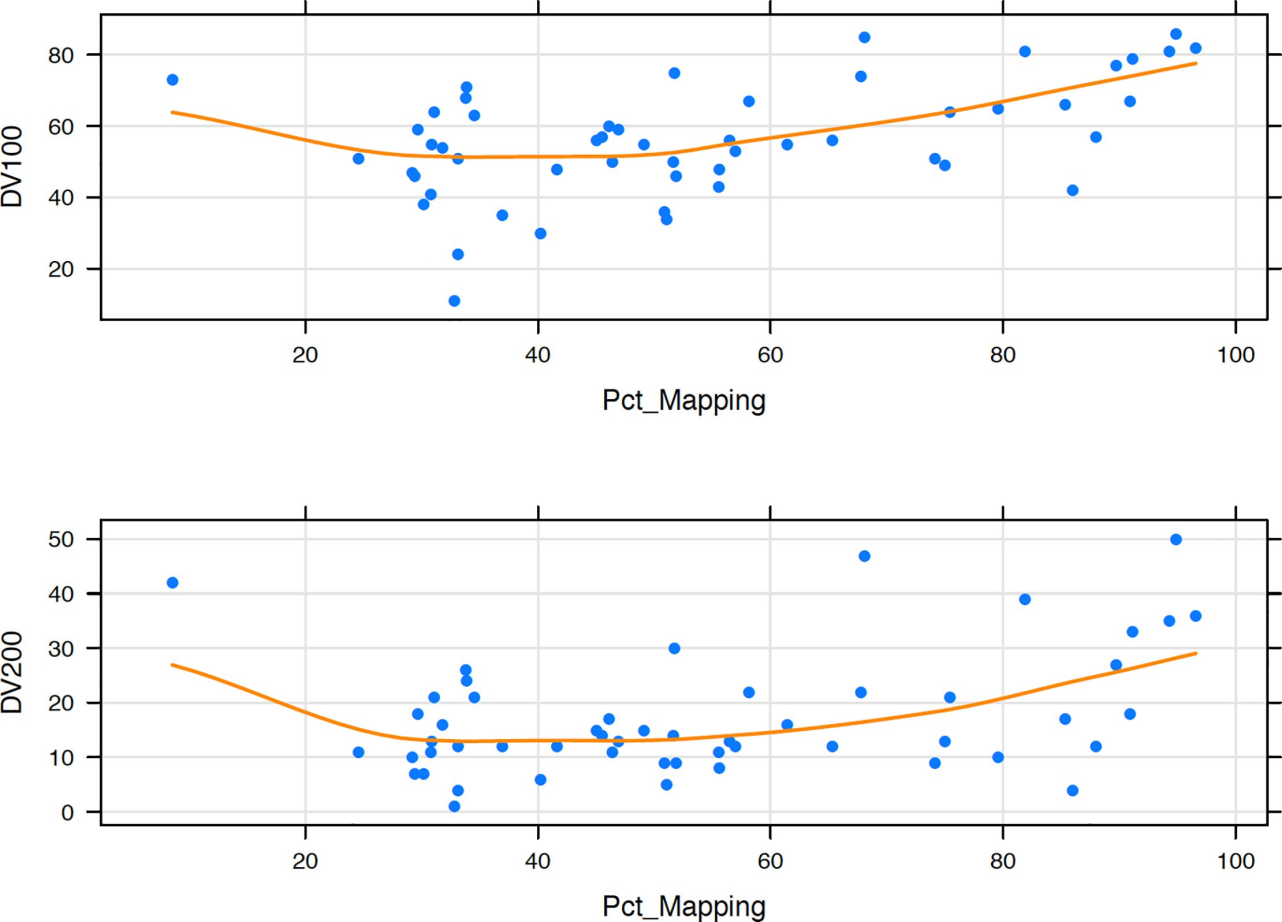
Percent mapped RNAs by DV_100_ and DV_200_ values. Plots showing percent mapped RNAs for samples with varying degrees of fragmentation, as depicted by fragment distribution values (DV_100_ (top plot) and DV_200_ (lower plot)). Every blue dot represents an individual sample, and the orange line represents the curve of best fit.

We also plotted DV_200_ and DV_100_ values against the percentage mapped RNA (Fig 3) in order to identify minimum requirements for the sample QC values. Though both DV_200_ and DV_100_ resulted in similar plots, DV_100_ provided greater resolution, making it the more suitable QC metric for highly degraded samples. Samples with DV_100_ below 40 resulted in poorer mapping of the sequenced RNA reads (percent mapping < 50), while samples with DV_100_ > 60 were more likely to perform better (percent mapping >60). With the caveat that DV value is not the sole determinant of sequencing outcome, we suggest, for future analyses of similar quality samples, proceeding with samples with DV100 > 40; we also suggest starting with higher input amounts of total RNA for all samples with DV100 < 60. The current Illumina recommendations for their TruSeq RNA Access / RNA Exome kit (the recommended kit for FFPE RNA samples) are based on DV_200_; values above 70% are considered good with input recommendations as low as 20 ng, while 20–50 ng and 50–100 ng are recommended for samples with medium (DV_200_ 50–70%) and low (DV_200_ 30–50%) quality. Illumina does not recommend proceeding with samples with DV_200_ < 30%. It is noteworthy that in the current set of samples, there were no samples with DV_200_ >50%, and only 8 had DV_200_ between 30% and 50% (Table 2 and Fig 3). Thus, our DV_100_-based recommendations are useful for samples that fall below the ‘low’ quality RNA, as defined by Illumina.

In order to further assess the quality of extracted RNA, we performed qPCR assays on a subset of SEER samples, along with some non-FFPE total RNA and some non-SEER FFPE RNA samples (S2 Table). We tested the panel of RNA samples for the presence of amplifiable transcripts for two housekeeping genes–*ACTB* and *HPRT1* (amplicon sizes 174 bp and 57 bp, respectively). The assay also included controls for the presence of inhibitors of reverse transcription and PCR amplification, and the presence of DNA contamination. We were unable to detect the presence of either of the two housekeeping genes in any of the FFPE-RNA samples tested, even though no inhibition of reverse transcription or PCR amplification was detected in any of the tested samples. The non-FFPE samples showed good amplification of both housekeeping genes. This indicates that the RT^2^ RNA QC PCR Array is not a good assay to assess the integrity of these samples. This could be either because of inherent incompatibility of some assay component(s) with FFPE samples, or because the genes or amplicons tested may not be optimum and another set of housekeeping genes / amplicons (with higher expression level in the assayed tissue and smaller amplicon size) may be more suited for FFPE-RNA.

Total RNA libraries were prepared from all the samples after rRNA depletion. Because the degraded FFPE-RNA fragments are likely to lack an intact polyA tail, rRNA depletion, followed by random priming during the reverse transcriptase reaction was chosen as the method of choice over the use of poly(dT) for mRNA selection and for priming first strand cDNA synthesis.

### QC measures of RNA sequencing

**Fig 4:**
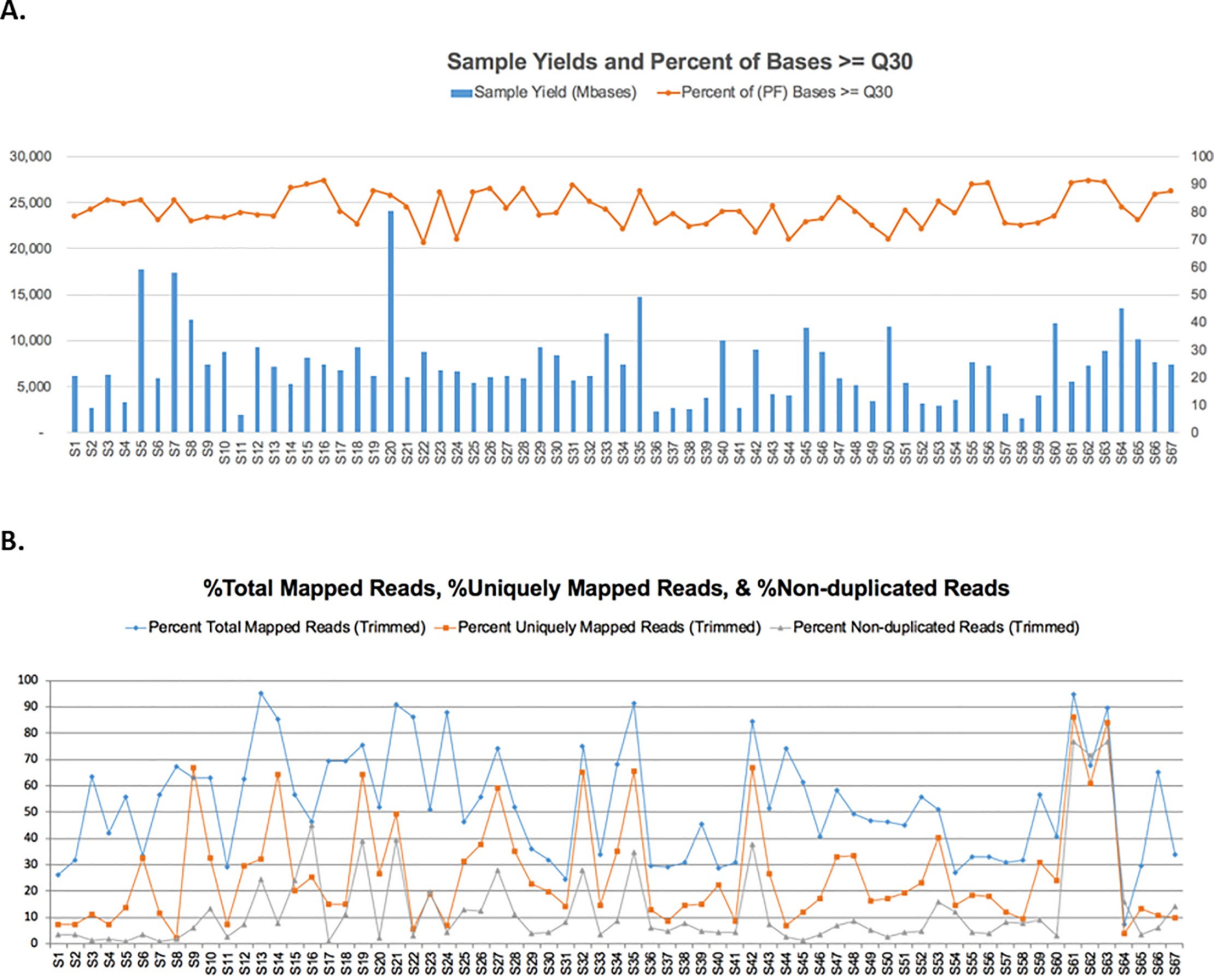
Sequencing QC Statistics. a) Sample sequencing yield and base call quality statistics. The bar chart shows the raw sequencing yields in million bases for each sample. There are large variations of the sequencing yield among the 67 samples in the study. The top line chart shows the percentages Q30 bases of all sequencing reads in each sample. b) Sample sequencing reads total mapped (% of reads that are mapped to reference genome), uniquely mapped (% of reads that are uniquely mapped to the reference genome) and non-duplicated reads (% of non-duplicated reads which are not PCR redundancy).

Several RNA sequencing QC parameters were examined (Table 2), including sequencing yield, Q30 (% of bases greater than Q30 is a metric for the overall quality and accuracy of base calling of the sequencing run [17]) (Fig 4A), % total mapped reads, % uniquely mapped reads, and % non-duplicated reads (Fig 4B). The total pass filter reads for all samples were between 10.6 million to 159.4 million due to large variation in the library quality. All samples resulted in 69–91% of bases greater than quality score of Q30 (Fig 4A). Sequencing reads from smaller fragment sizes have very short portions of useable reads and are more likely to have ambiguous alignments to the reference genome, and are thus filtered out by the mapping software. Therefore, highly degraded RNA samples have very low mapping rates, which is reflected in the positive correlation between percent total mapping and DV values, e.g., a correlation value of 0.58 with DV_100_ (Fig 2). Percent total mapping to the human genome (hg38) was between 7.4% to 94.9% (Fig 4B). Percent unique mapping was between 2% to 86% and percent non-duplicated reads for the samples ranged from 1% to 76%. These QC measures indicate vast differences in the amount of unique fragments per sample.

### RNA statistics and potential artifacts

Picard’s CollectRNAStatistics was used to determine the percent ribosomal RNA, mRNA, intronic, and intergenic bases present in the alignment files. All samples had less than 2.5% ribosomal bases. Intronic bases varied between 17% to 72%. Intergenic bases varied between 11% to 81%. The percentages of mRNA bases were between 1% to 34% (S1 Fig).

**Fig 5:**
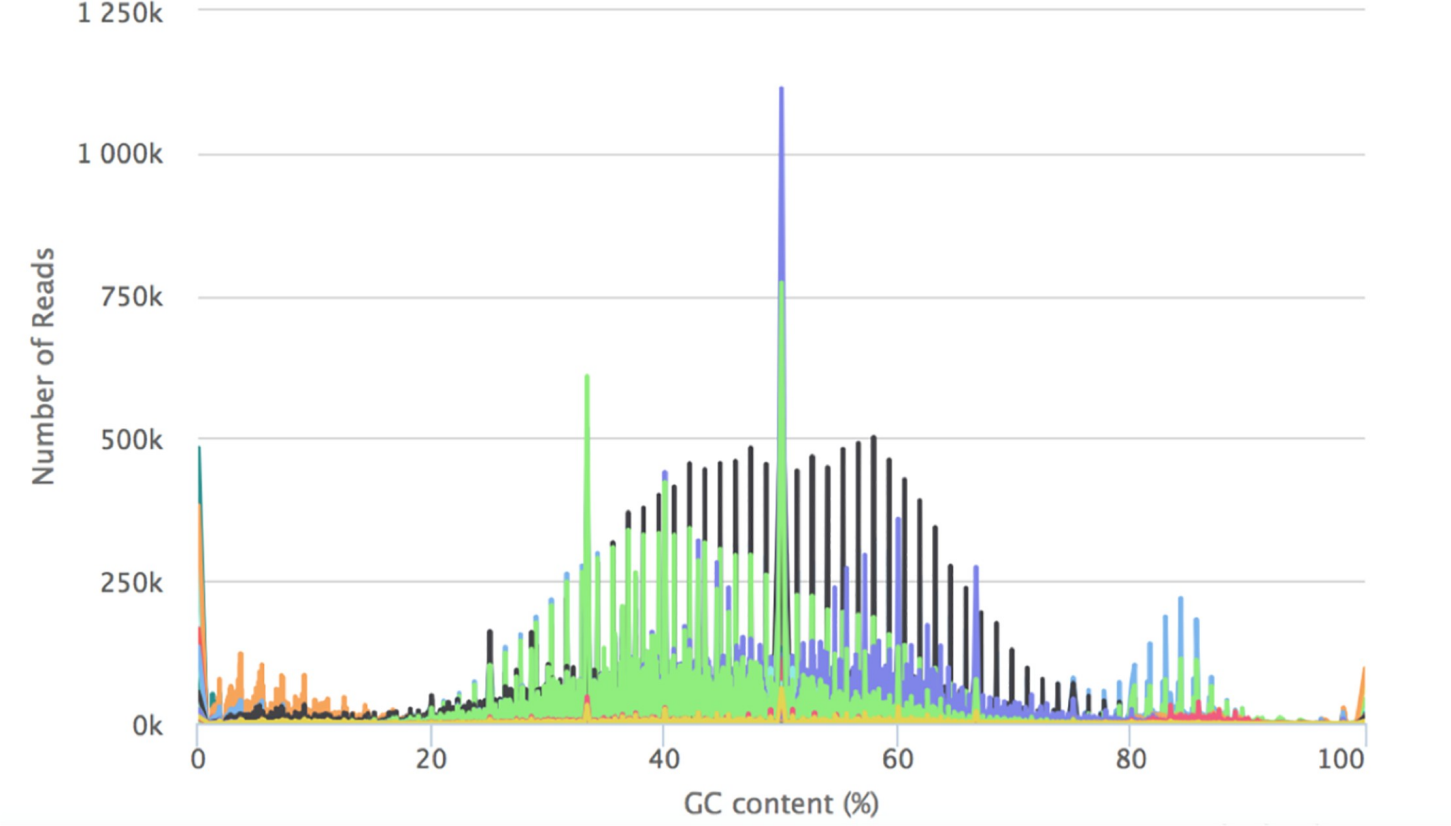
All sample GC content versus number of reads. The plot shows the GC distribution scaled to 100 bins. The vertical bars with the same color denote the GC distributions for the same sample.

In terms of the GC content profiles, some samples showed abnormal peaks around 5% and 85% GC content (Fig 5). The extremely low and high GC samples have very low mRNA mapped reads compared to samples with balanced GC content (the GC content normally is in range of 40%-60% for human samples). This observation agrees with a previous study [18], where high GC FFPE samples had a large fraction of reads mapped to the intronic regions that may have contributed to the abnormal GC-content peak in FFPE samples. The FFPE fixation process is known to introduce molecular artifacts such as GC content alterations compared with fresh frozen tissues [4].

**Fig 6:**
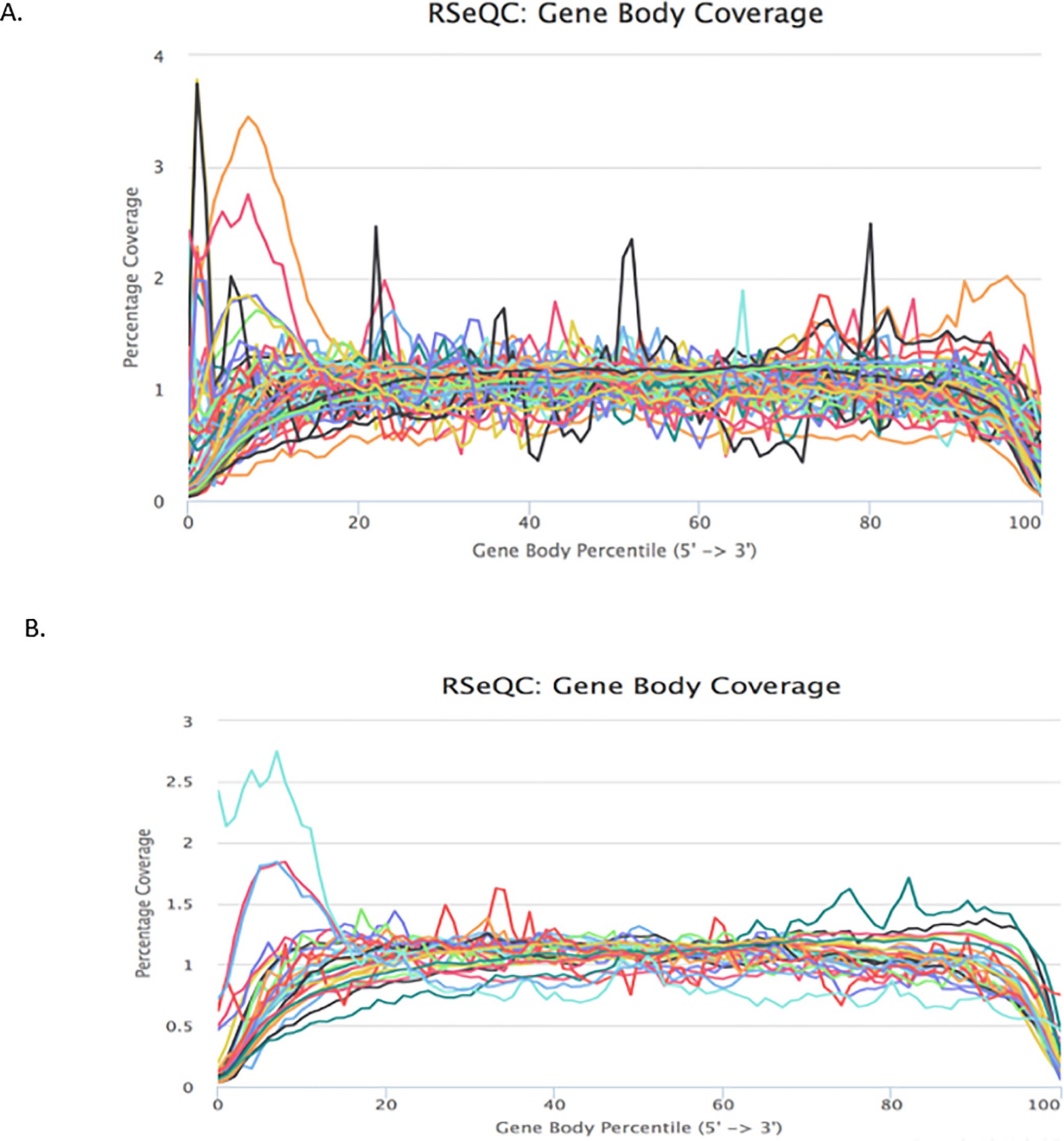
Gene body coverage plots. a) All samples. b) Samples with mRNA percentage greater than 10.

Gene Body Coverage plots measure the percentage of reads that cover each nucleotide position of all of genes scaled to 100 bins from 5′ UTR to 3′ UTR. It is used to identify 3’ or 5’ bias that may have occurred during the library preparation or sequencing process. A 3′-end bias in gene body coverage may indicate degradation of RNA. A 5′-bias may be due to alternative poly-adenylation sites [18]. The coverage plot over normalized gene body for all samples indicates a small set of samples having either 5’ bias, or 3’ bias (Fig 6A). The rest of the samples have relatively uniform coverage distributions. In order to assess whether sample quality and mRNA content may play a role in the 5’ and 3’ bias for gene body coverage, we split the samples into two groups based on the mRNA yield. We then selected 32 samples that had mRNA percentages greater than 10% and observed that the bias along the transcript length was mostly eliminated from the higher quality RNA sample group (Fig 6B). This result is consistent with previous studies [19, 20] that revealed ribosomal RNA depletion based protocols provided more uniform transcript coverages than poly-A selection based mRNA-Seq protocol. However, we found that with highly degraded RNAs, 5′ to 3′ coverage bias along transcripts were not completed eliminated.

We also investigated the sample storage age impact on the uniformity of the read coverage by calculating the correlations between the specimen storage time with the read mapping rates for the exonic, intronic regions as well as the uniformity of the read coverage depicted by the 5’ to 5’ bias. There were slightly negative correlations (S2 Fig).

### Contamination assessment

Fifty six of the 67 samples were found to have greater than 1% bacterial contamination (Table 2). 41 of these had greater than 5% bacterial contamination. A common species contaminating the samples was *Propionibacterium acnes*. Although all of the contamination of the samples cannot be explained by this species, up to 14% of the total reads accounted for in the contaminated samples were from this bacteria, with percentages ranging from 0.02% to 14.35% of the reads. Twenty-eight of the samples had more than 5% of reads mapping to *Propionibacterium acnes* (S3 Fig).

There were six samples contaminated with mouse genome with 4%–11% of reads mapped to mouse genome. Since the six samples were from two different SEER sites, not all samples from the same sites were contaminated. In addition, among the six contaminated samples, one sample had a biological replicate (sample SEER_045 and sample SEER_065) collected from same patient and stored at the same SEER site; only SEER_065 had reads mapped to mouse genome in the 4–11% range. This suggests that samples were not contaminated during the initial sample collection, but may have been introduced in somewhere along the sample preparation process (which includes all of the steps from tissue acquisition through to RNA sequencing). While we cannot conclude where the contamination was from, we do know that contamination with the bacterial or mouse genomes was not from a single SEER site; rather, it was found in 56 samples from all 3 SEER sites.

### Specimen storage time, genome mapped reads and mRNA correlations

The correlation analysis (Fig 2) between specimen storage (archival) time and total percentage of mapped reads showed a weak negative correlation value of -0.14, which suggests that the storage time might affect the sample quality. In addition, the bioanalyzer library molarity and mapped mRNA reads shows a positive correlation value of 0.37. We have observed high percentages of the mapped reads aligned to intergenic regions as well as intronic regions, especially for poor quality samples with extremely low or high GC content. The DV_100_ has positive correlations with the percentage of reads mapped on human genome (0.58), non-duplicated reads (0.53), as well as total gene detected (0.43). DV_100_, together with bioanalyzer library molarity values after library prep, can be used as a metrics parameter to screen poor quality samples before sequencing.

**Fig 7:**
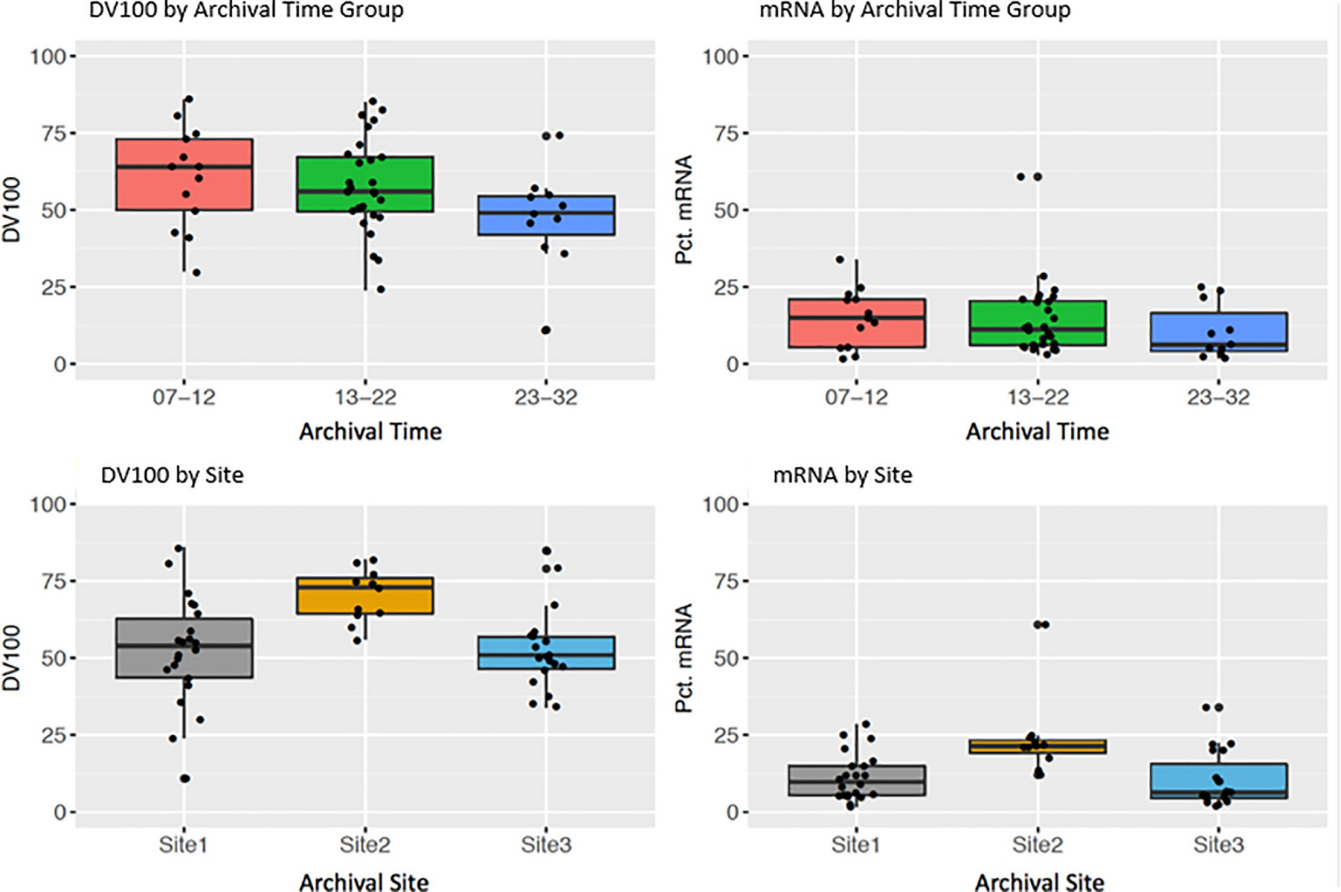
Sample qualities, specimen storage time (i.e. archival time) and SEER site correlations. Box plots showing the sample quality distribution measured by DV100 and mRNA content in 3 specimen archival time groups (upper plots); box plots showing the sample quality distribution measured by DV100 and mRNA content in 3 SEER archival sites (lower plots).The box represents first and third quartiles intersected by median value. The whiskers indicate the maximum and minimum values of the range. Each sample was represented as a dot in the plot.

In order to better depict the relationship between sample storage time and sample quality, we divided the samples into three storage time cohorts, and calculated the mean value and SD of the sample DV_100_ and sample mRNA content. The longer storage time was associated with lower RNA quality (DV_100_) and lower mRNA content from NGS analysis. However, no significant correlation was observed between SEER site and DV_100_ or mRNA content (Fig 7).

We further investigated which gene group might be conserved among samples across different specimen storage time cohorts and SEER sites based on gene expression profiles. We extracted 189 genes that had relatively high expression across samples (Part A of S3 Table). KEGG Pathway analysis of the 189 genes showed most of them were related to Ribosome, P13K-Akt signaling pathway, ECM-receptor interaction and Focal adhesion pathways (Part B of S3 Table). The P13K-Akt signaling pathway is known as a central regulator of ovarian cancer. This result suggests that valuable gene expression information could be extracted from aged FFPE blocks with degraded RNAs based current NGS technologies.

### Correlations between biological replicates

Seven pairs of biological replicates (2 separate sets of tissue curls were cut from the same FFPE blocks for 7 subjects) were included in this study (S4 Table). The sample QC assessment showed most replicates (6 out of 7) had very similar RINs. However, the two oldest pairs of the samples (SEER 005 (24 year old sample) and SEER 007 (18 year old sample)) each had one sample with extremely low RIN value around 1.0. The library yields for the replicated samples showed from 1 to 100-fold difference between replicates. In addition, 5 pairs of replicates were heavily contaminated with bacteria genomes but the percentages of bacterial contamination between the replicates were very different, suggesting sample contamination. Moreover, the sequencing yields between the replicates were also different. Among the 7 pairs of replicates, 4 pairs of biological replicates had correlation values between 0.7–0.8 when comparing gene expression values between the replicates pairs; the remaining 3 pairs had correlations below 0.22. The low correlations for the biological replicates indicate that the variability in extracted RNA and sample processing have contributed towards the differences in sample gene expression profiles. Principal Components Analysis (PCA) showed 3 samples were extreme outliers while rest of the 11 samples are more closely related based on gene expression (S4 Fig). For some of the samples with similar gene expression profiles, the correlations were above 0.85, indicating that the results among high quality samples including biological replicates are reproducible.

### TCGA ovarian prognostic signature gene set expression profile

In order to compare our sequencing data with pre-existing ovarian cancer data, we selected TCGA ovarian prognostic signature gene set [21] which includes 193 genes expressed in a fashion that can predict patient survival; TCGA data were generated using high-quality fresh-frozen tissues. We profiled the expression level of this gene set using the SEER FFPE RNA-Seq data. All of the 193 genes from the TCGA dataset were found in the SEER FFPE RNA-Seq data set with a minimum of 5 read counts per gene as the minimum cutoff threshold. The gene expression values had a wide dynamic range with up to nearly 100 fold change differences among the 193 ovarian prognostic signature gene set.

**Fig 8:**
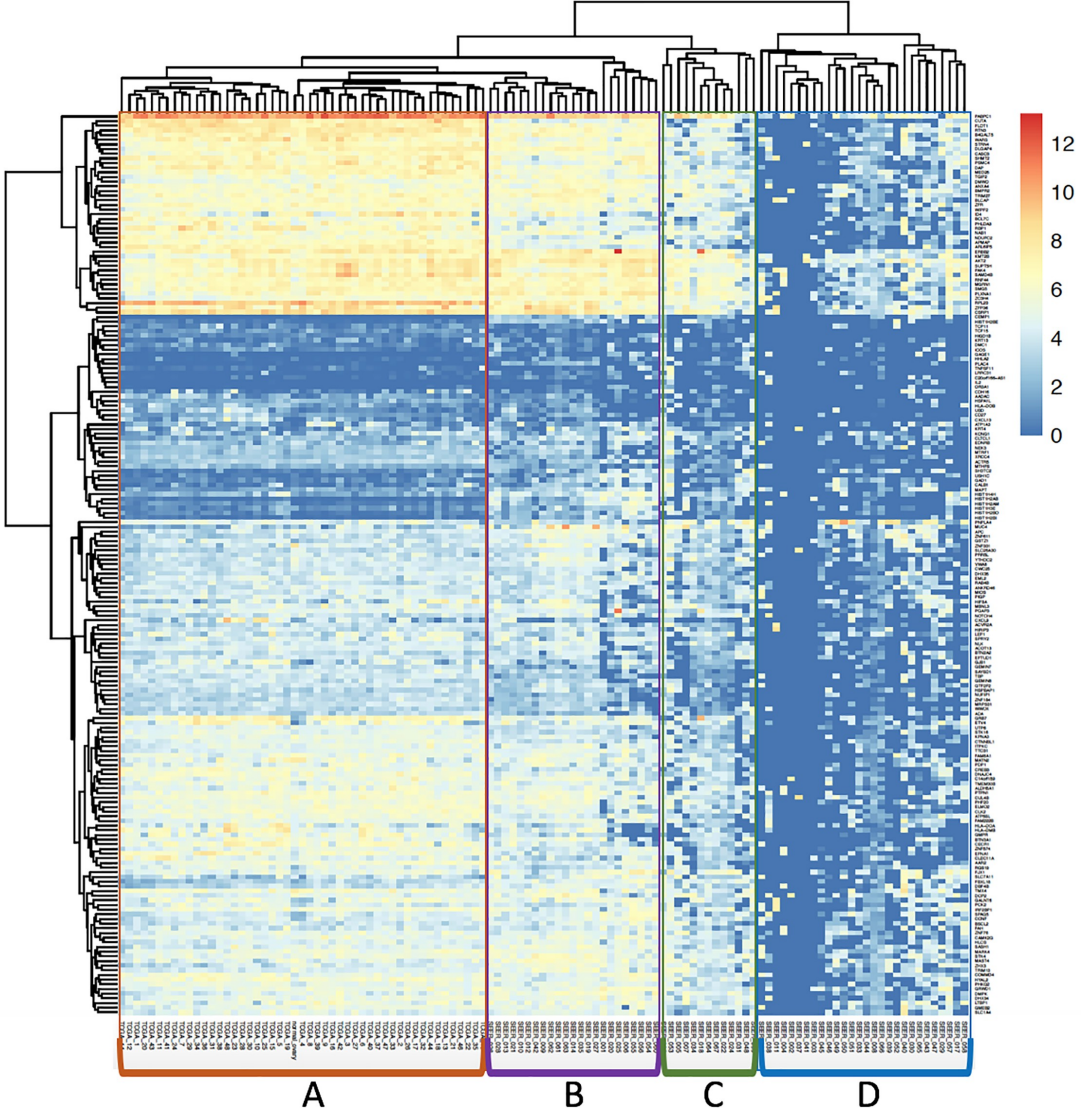
TCGA ovarian prognostic signature gene set expression profile heat map. Cluster heat maps of 193 ovarian prognostic signature gene profile among 67 SEER FFPE samples, 48 TCGA ovarian tumor samples and one Human BodyMap normal ovary tissue sample. Each row represents a gene and each column represents a sample. The color and intensity of the boxes represent changes of gene expression. Genes and samples have been hierarchically clustered based on their gene expression pattern. Group A samples contain the 48 TCGA ovarian tumor samples and one Human BodyMap ovary tissue sample. Group B samples are the 26 SEER samples which display a good correlation with TCGA ovarian tumor samples. Group C samples are the 13 SEER samples which have low to medium overall gene expression of the 193 ovarian prognostic signature gene set. Group D samples contain the rest of the 28 SEER samples with very low overall gene counts.

In addition, we downloaded 48 RNA-Seq data sets from TCGA Ovarian Serous Cystadenocarcinoma project (https://portal.gdc.cancer.gov/projects/TCGA-OV) and one normal ovary tissue RNA-Seq data set from Human BodyMap Illumina project (https://www.ebi.ac.uk/arrayexpress/experiments/E-MTAB-513). We then examined gene expression profiles of the 193 signature gene panel using the three data sets. The expression level was higher overall in a majority of the 193 genes in the TCGA fresh-frozen ovary tumor samples and Human BodyMap normal ovary tissue data sets compared to the SEER FFPE data set. This may be due to higher sequencing depth in the TCGA/Human BodyMap data sets than SEER FFPE RNA-Seq data. While SEER FFPE samples were targeted for 50 million reads per sample, TCGA-OV data set had 100–300 million reads per sample and Human BodyMap ovary tissue sample had about 243 million reads. Among the 67 SEER FFPE RNA-Seq samples, 26 SEER samples (Fig 8, cluster B) showed good correlation with TCGA and the Human BodyMap data sets (Fig 8, cluster A) for the majority of the genes. 13 SEER samples (Fig 8, cluster C) displayed low to medium overall expression of the 193 signature gene set. 28 SEER samples had very low overall expression and clearly were clustered as completely different clusters (Fig 8, cluster D). Most of those in cluster D had very low library yields and molarity compared to the rest of the samples. For these 28, there was low quantity of cDNA to sequence, very few mapped mRNA reads and very low gene counts.

Among the 193 prognostic signature gene set, we observed the gene expression level are different between the TCGA cluster A samples from the rest of the SEER samples. Such differences could be the consequence of different rRNA depletion methods as well as RNA extraction kits used. We also observed only a small subset of genes were expressed in SEER Cluster B but missing in TCGA Cluster A data sets. Upon closer examination, we found that those genes encode histone proteins. The transcripts of the histone genes, such as *HIST1H2AB*, *HIST1H2BE* or *HIST1H3E*, lack polyA tails, therefore, the polyA capture based RNA-seq protocol could miss those transcripts in TCGA data set.

## Discussion

This study demonstrates that FFPE samples acquired from SEER under varying storage duration and conditions still have potential value as sources of RNA for NGS studies. Longer FFPE tissue storage time was associated with more degradation of the RNA (which supports previous findings [6, 22]). This in turn can cause lower yields in library product, as we have shown. For a large portion of the samples (60%), gene expression quantification found more than 10,000 genes; the number of genes expressed was comparable to human control RNA (about 13,000 genes). In addition, the correlations among 4 out of 7 biological replicates were above 0.70.

Our study complements and expands upon several published studies that examined the use of FFPE tissues for RNAseq. As noted in a recent review article on the use of FFPE for high throughput genomic studies, reliable gene expression data can be obtained from RNAseq using FFPE tissues, provided they are not stored for a long periods of time [22]. Our study expands upon the storage time issue by including a wider range of storage times. We examined the use of FFPE tissues that had been stored up to 32 years (which was 12 years longer than samples analyzed in previous studies [6, 22]) and found that while storage time was negatively associated with RNA sample quality, gene expression data could be obtained regardless of specimen storage time. Additionally, while traditional QC measures such as RIN are often used, they rarely predict the success of sequencing from FFPE tissues [23]. Here, we suggest using DV_100_ for initial sample QC, combined with library quality analysis using bioanalyzer and qPCR to avoid sequencing of samples that are not likely to yield useful data. We further expanded upon previous studies by using a larger number of FFPE tissues from a single cancer site. Our study leveraged the SEER registries to obtain FFPE tissues from 60 ovarian cancer cases, which was more than triple what previous studies used for examining the utility of FFPE for RNAseq using a single cancer site; these previous studies had used between two [7–10, 18, 24] to 19 FFPE tissues from a single cancer site [6]. Together, the large sample size and wide range of specimen storage times allowed a more robust development of quality metrics for helping to predict RNAseq success and study design elements to increase the utility of resulting RNAseq data from archived FFPE tissues.

Another strength of our study was that the samples are reflective of what epidemiologists might anticipate having access to for translational clinical research studies. Residual tissues from surgical resections are typically preserved as FFPE blocks, and subjected to a variety of storage conditions. Our study shows that it is not essential to have pristine FFPE blocks in order to conduct RNA-seq analyses and obtain useable results.

Sample quality was one of the limitations of our study. We found that 40% of the samples had very low mRNA and gene counts due to highly degraded RNAs, and about 80% of the samples were contaminated with bacterial or mouse RNA. Larger library size was correlated with a lower percentage of total mapped reads to the human reference sequence (-0.78). Samples with heavier bacterial and mouse contamination had larger library sizes (correlation value is 0.77), which can explain the lower mapping percentages. As previous studies [19, 25] suggested, targeted exome capture transcriptome protocols, such as Illumina TruSeq RNA Access Library Prep / RNA Exome kit, designed to capture the target gene regions using capture probes, effectively eliminated the contaminating species from costly sequencing and provided accurate and unbiased estimates of RNA abundance for degraded RNAs. However, the TruSeq RNA Access kit is not recommended for highly degraded samples (DV_200_ < 30%) based on the protocol. Only 8 of the 53 SEER FFPE samples for which we could measure DV_200_, had DV_200_ > 30%. Therefore, initial QC checks for RNA degradation and selection of a suitable library preparation protocol based on the RNA degradation profile is highly recommended. Our analysis showed that ribosomal RNA depletion based whole transcriptome analysis protocol such as NEBNext Ultra II RNA Library Prep Kit for Illumina works very well with sample input far lower in quantity and quality than typically recommended. However, it still showed limitations for handling highly degraded RNA samples. This observation is consistent with the recent study by Schuierer and colleagues [19]. Careful experimental design and bioinformatics analyses are critical to ensure high accuracy and reproducibility for profiling of very heterogeneous RNA samples that cover a wide range of low quantity and extremely low quality samples.

From our study, we observed that gene body coverage along the transcript length was fairly even for a majority of the RNA samples. Data from this study agree with a previous study done by Graw and colleagues [18] that random priming during reverse transcription of RNA from FFPE tumor samples effectively eliminates the 3′ bias expected from the heavily degraded RNA. Thus, we recommend rRNA depletion using RNase H and random priming of the reverse transcription reaction for preparing sequencing libraries from FFPE-RNA samples, over the use of poly(dT) for mRNA selection and conversion to cDNA.

Another limitation to this study was the lack of fresh frozen (FF) tumor tissue from the same patients to compare the results. However, several studies have provided evidence showing FFPE as an acceptable alternative when FF tissue is not available for NGS [6, 8, 9, 18, 20, 24, 26]. From our study, we extracted genes that are relatively highly expressed among samples from different specimen storage time cohorts and storage sites and performed pathway analysis. Most of the genes are related to the Ribosome, P13K-Akt signaling pathway, ECM-receptor interaction and Focal adhesion pathways. The P13K-Akt signaling pathway is known as a central regulator of ovarian cancer. Moreover, we profiled the selected 193 ovarian prognostic signature gene set among the SEER FFPE samples, FF Ovarian Serous Cystadenocarcinoma sample from TCGA-OV project RNA-Seq, and the FF normal ovary tissue sample from Human BodyMap Illumina RNA-Seq project. The result of profiling the 193 ovarian prognostic signature gene showed 26 of the 67 SEER samples (39%) had similar expression patterns to the TCGA datasets despite the wide range of variations in storage time, sequencing depth, sample quality and library preparation protocol differences. Twenty eight (42%) of the SEER samples had low gene counts. The low gene counts were not associated with storage time. The low gene counts were likely due to very poor sample quality and extremely low library yields and library molarity, which limited the amount of cDNA available for sequencing and resulted in very low number of mapped mRNA reads.

The results collectively suggest that the NGS data produced from the FFPE SEER archive tissue can be reproducible when samples meet specific quality control criteria or the following caveats are taken into account:

- Sample quality assessment using the normal QC metrics such as DV_200_ or RIN alone may not provide an accurate measurement for highly degraded RNA. This is in agreement with several earlier studies that showed RIN may not be an accurate RNA quality assessment measure [23]. Therefore, it is necessary to adjust the QC metrics to assess shorter RNA fragments.
- DV_200_ is not very informative for highly degraded samples, but DV_100_ provides some resolution to assess the sample quality. Based on this study of FFPE-RNA, we suggest proceeding with samples with DV_100_ > 40. For all samples with DV_100_ < 60, it is beneficial to use higher sample input amounts, whenever possible. Using DV_100_ for initial sample QC, combined with library quality analysis using bioanalyzer and qPCR are often necessary to avoid sequencing samples that are not likely to yield useful data.
- Anticipate more degradation of the RNA in older FFPE tissues. However, RNA-Seq can still yield reliable gene expression data for degraded RNA. Due to FFPE having relatively heavily degraded RNA, we suggest rRNA depletion using RNase H and random priming of the reverse transcription reaction for preparing sequencing libraries from FFPE-RNA samples, over the use of poly(dT) for mRNA selection and conversion to cDNA
- Assess potential contamination of the RNA as part of the standard post sequencing RNA-Seq QC protocols. Bacterial contamination and genomic DNA contamination are common for some of the FFPE samples independent of storage condition of sites and sample preparation procedures. Contamination can lead to misinterpretation of results, especially when working with other species (such as the microbiome). A recent article by Heintz-Buschart et al [27] showed contamination with non-human sequences could be traced to RNA extraction columns; we did not use a control RNA extraction (with no sample) to control for contamination introduced during the RNA purification, but this may be a good precaution to take for future studies. Highly contaminated samples that do not yield much mRNA from the original tissues should be excluded from further downstream data analysis.
- Gene expression data from samples that have low quality cDNA, few mapped mRNA reads, and generate low gene counts should be used with caution. In our study, expression levels of the 193 signature gene panel in the 28 SEER samples with low gene counts (Fig 7, Cluster D) did not correlate well with TCGA, suggesting the RNAseq data from low gene count samples is not of good quality for gene expression analyses.

## Conclusions

Recent studies have shown remarkably high consistency between RNA-seq data generated from paired freshly frozen and FFPE tissue samples [6, 8, 9, 18, 20, 23, 24, 26]. Our study provides additional evidence and suggestions for feasibly conducting gene expression analysis using RNA-Seq with FFPE tissues spanning a wide range of storage times. There is no denying that there are technical and quality limitations for FFPE RNA-Seq data. However, many of these issues can be overcome through thorough QC and thoughtful bioinformatics analyses. Our study supports the notion that RNA-Seq on archived FFPE samples can be used as an unbiased and comprehensive assessment of gene expression in biomedical studies.

## Supporting information

S1 Fig RNA mapping based on genome gene annotation. (TIFF)

S2 Fig Specimen storage time (age of sample) correlation with read mapping on coding, UTR, intronic and uniformity of read coverage. (TIFF)

S3 Fig Sample Contamination Assessment—Mapping to Bacteria species and Mouse. (TIFF)

S4 Fig PCA analysis for biological replicates. PCA biplot with samples plotted in two dimensions using their projections onto the first two principal components. Each pair of replicates have color keys displayed on the upper right legend.
(TIFF)

S1 Table Analysis Software Version and Parameters. (DOCX)

S2 Table qPCR assay results on a subset of SEER samples, along with some non-FFPE total RNA and some non-SEER FFPE RNA samples. (DOCX)

S3 Table **A:** Conserved genes in samples across specimen storage times and SEER sites. **B:** KEGG Pathway of 189 Highly Expressed Genes.
(DOCX)

S4 Table Biological Replicates QC and Sequencing Statistics. (DOCX)
