# Supplementary figures and images for: Robustness of RNA sequencing on older formalin-fixed paraffin-embedded tissue from high-grade ovarian serous adenocarcinomas
Source: PLoS One. 2019 May 6;14(5):e0216050. doi: 10.1371/journal.pone.0216050 (PMC6502345; doi:10.1371/journal.pone.0216050)

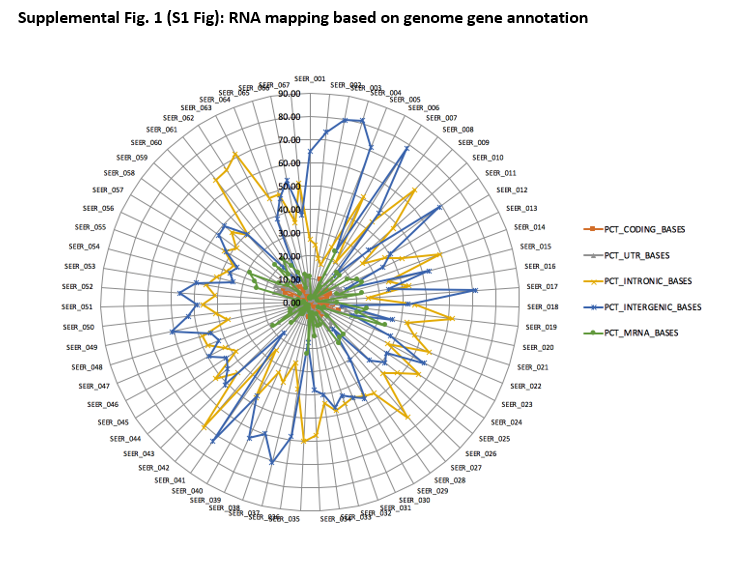

Supplement: S1 Fig — (TIFF) [file pone.0216050.s001.tiff]

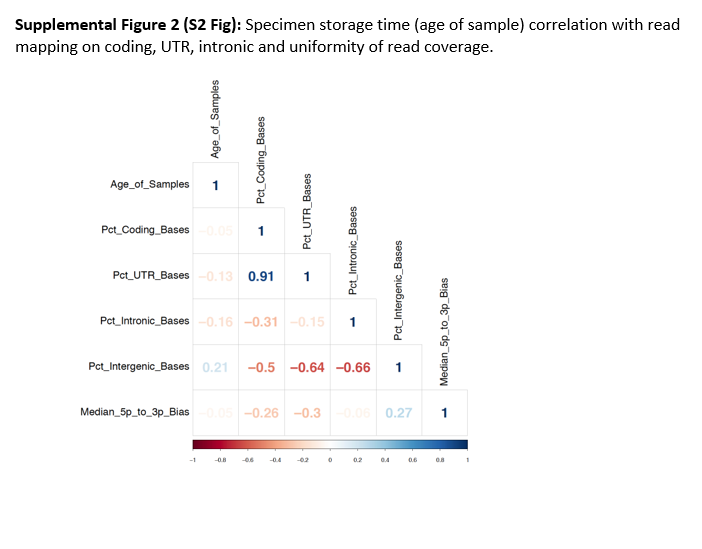

Supplement: S2 Fig — (TIFF) [file pone.0216050.s002.tiff]

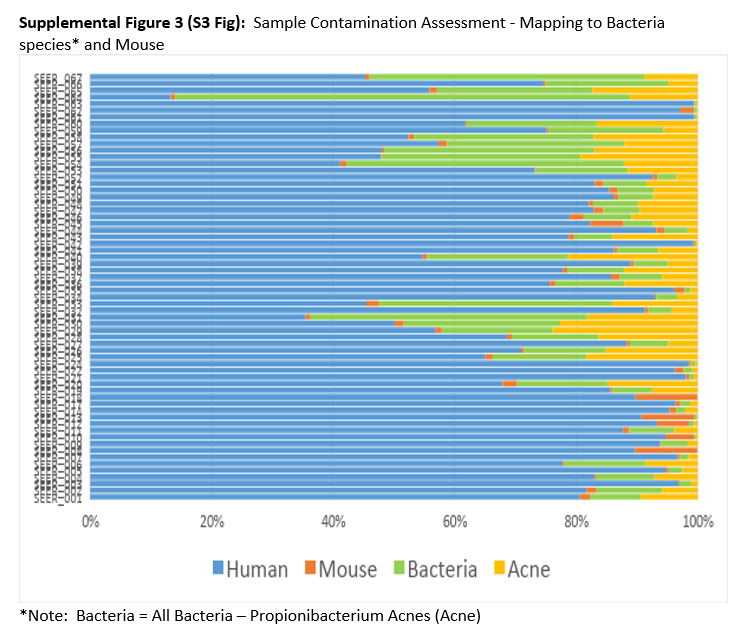

Supplement: S3 Fig — (TIFF) [file pone.0216050.s003.tiff]

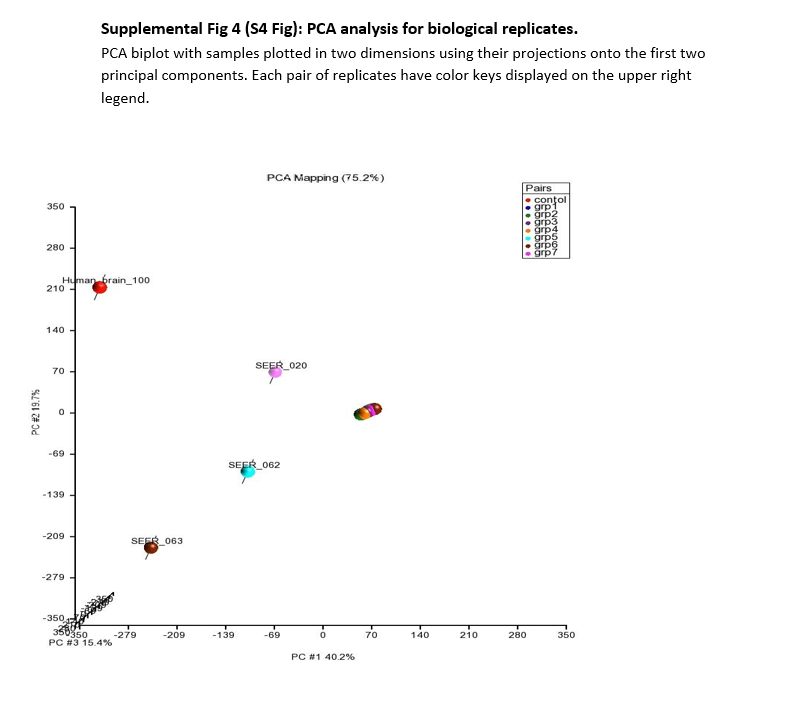

Supplement: S4 Fig — PCA biplot with samples plotted in two dimensions using their projections onto the first two principal components. Each pair of replicates have color keys displayed on the upper right legend. (TIFF) [file pone.0216050.s004.tiff]
