# Supplementary material for: Robustness of RNA sequencing on older formalin-fixed paraffin-embedded tissue from high-grade ovarian serous adenocarcinomas
Source: PLoS One. 2019 May 6;14(5):e0216050. doi: 10.1371/journal.pone.0216050 (PMC6502345; doi:10.1371/journal.pone.0216050)
Supplement: S1 Table — (DOCX) [file pone.0216050.s005.docx]

**S1 Table: Analysis Software Version and Parameters:**

| **Analysis Step** | **Software** | **Software Parameters / Notes** |
| --- | --- | --- |
| Basecalling | RTA 1.18.66.3 | Illumina instrument run time analysis software |
| Demultiplexing | Bcl2fastq 2.17 | Barcode demultipling and allowing 1 mismatch in barcodes |
| Filtering (Adaptor and quality) | Trimmomatic 0.30 | PE -threads 16 -phred33 ILLUMINACLIP:adapters.fa:2:36:10 LEADING:10 TRAILING:10 MAXINFO:50:0.97 MINLEN:20 |
| Alignment | STAR 2.5.1 | --genomeDir $star_genome --readFilesIn R1_trimmed.fastq R2_trimmed.fastq --outTmpDir /logs/star --outSAMunmapped Within --outFilterType BySJout --outFilterMultimapNmax 20 --outFilterMismatchNmax 999 --outFilterMismatchNoverLmax 0.04 --alignIntronMin 20 --alignIntronMax 1000000 --alignMatesGapMax 1000000 --alignSJoverhangMin 8 --alignSJDBoverhangMin 1 --sjdbScore 1 --runThreadN 8 --genomeLoad NoSharedMemory --outSAMtype BAM Unsorted --quantMode TranscriptomeSAM |
| RNAStatistics | Picard 1.84 | CollectRnaSeqMetrics.jar REF_FLAT=annotation_refFlat.txt INPUT=sample.bam OUTPUT= RnaSeqMetrics.txt RIBOSOMAL_INTERVALS= ribosome_interval_list.txt STRAND_SPECIFICITY=NONE VALIDATION_STRINGENCY=LENIENT |
| Duplication Statistics |  | MarkDuplicates.jar INPUT=sample.bam OUTPUT=sample.MKDUP.bam METRICS_FILE=sample.bam.metric ASSUME_SORTED=true MAX_FILE_HANDLES_FOR_READ_ENDS_MAP=1000 VALIDATION_STRINGENCY=LENIENT |
| Insert Size Statistics | RseQC 2.3.5 | inner_distance.py–i sample.bam –o ./ -r annotation.bed  infer_experiment.py $rseqc_file –i sample.bam  read_GC.py –i sample.bam  read_distribution.py –i sample.bam  junction_saturation.py –i sample.bam –r annotation.bed |
| Quantification | RSEM 1.2.22 | rsem-calculate-expression –bam --paired-end --estimate-rspd Transcriptome.out.bam $RSEM_Genome $Sample_Name |
