# Supplementary material for: Robustness of RNA sequencing on older formalin-fixed paraffin-embedded tissue from high-grade ovarian serous adenocarcinomas
Source: PLoS One. 2019 May 6;14(5):e0216050. doi: 10.1371/journal.pone.0216050 (PMC6502345; doi:10.1371/journal.pone.0216050)
Supplement: S2 Table — (DOCX) [file pone.0216050.s006.docx]

**S2 Table :** qPCR assay results on a subset of SEER samples, along with some non-FFPE total RNA and some non-SEER FFPE RNA samples.

| **Sample Name** | **DV_200_** | **DV_100_** | **Amount for QC (ng)** | **qPCR threshold cycle (C_T_) value** | | | | | | | |
| --- | --- | --- | --- | --- | --- | --- | --- | --- | --- | --- | --- |
|  |  |  |  | **HK1 = ACTB** | **HK2 = HPRT1** | **RTC** | **PPC (cDNA)** | **GDC** | **NRT** | **PPC (H_2_O)** | **NTC** |
| **Total_RNA_1** | NA | NA | 500 | 23.0 | 26.3 | 23.8 | 21.0 | Neg | Neg | 21.2 | Neg |
| **Total_RNA_2** | NA | NA | 500 | 22.6 | 25.9 | 23.9 | 21.0 | Neg | Neg | 21.2 | Neg |
| **Total_RNA_3** | NA | NA | 500 | 21.8 | 26.0 | 24.0 | 20.8 | Neg | 34.4 | 21.2 | Neg |
| **Total_RNA_4** | NA | NA | 500 | 23.8 | 26.9 | 23.6 | 20.9 | Neg | 34.1 | 22.2 | Neg |
| **Total_RNA_5** | NA | NA | 500 | 24.6 | 30.6 | 23.6 | 20.8 | Neg | 28.0 | 21.9 | Neg |
| **FFPE_RNA1** | 5 | NA | 500 | Neg | Neg | 23.8 | 20.7 | Neg | 30.7 | 22.0 | Neg |
| **FFPE_RNA2** | 1 | NA | 500 | Neg | Neg | 23.7 | 20.9 | Neg | 33.3 | 21.6 | Neg |
| **FFPE_RNA3** | NA | NA | 500 | Neg | Neg | 23.7 | 21.0 | Neg | 33.8 | 21.4 | Neg |
| **SEER_013** | 35 | 81 | 500 | Neg | Neg | 23.1 | 20.9 | Neg | Neg | 21.7 | Neg |
| **SEER_018a** | 36 | 82 | 500 | Neg | Neg | 23.0 | 20.8 | Neg | Neg | 21.2 | Neg |
| **SEER_018b** | 36 | 82 | 100 | Neg | Neg | 22.4 | 20.4 | Neg | Neg | 20.9 | Neg |
| **SEER_018c** | 36 | 82 | 100 | Neg | Neg | 22.5 | 20.5 | Neg | Neg | 20.8 | Neg |
| **SEER_018d** | 36 | 82 | 25 | Neg | Neg | 22.5 | 20.4 | Neg | Neg | 21.0 | Neg |
| **SEER_019** | 21 | 64 | 500 | Neg | Neg | 23.5 | 20.8 | Neg | Neg | 22.4 | Neg |
| **SEER_021** | 18 | 67 | 500 | Neg | Neg | 23.6 | 21.0 | Neg | Neg | 22.7 | Neg |
| **SEER_041** | 11 | 41 | 500 | Neg | Neg | 23.5 | 20.9 | Neg | Neg | 22.4 | Neg |
| **SEER_042** | 39 | 81 | 500 | Neg | Neg | 22.9 | 20.7 | Neg | Neg | 21.2 | Neg |
| **SEER_043** | 14 | 50 | 500 | Neg | Neg | 23.0 | 20.9 | Neg | Neg | 21.2 | Neg |
| **SEER_045** | 16 | 55 | 500 | Neg | Neg | 23.0 | 20.8 | Neg | Neg | 21.2 | Neg |
| **SEER_050** | 13 | 59 | 500 | Neg | Neg | 23.1 | 20.7 | Neg | Neg | 21.2 | Neg |
| **SEER_051** | 15 | 56 | 500 | Neg | Neg | 23.1 | 20.8 | Neg | Neg | 21.2 | Neg |
| **SEER_054** | 7 | 46 | 500 | Neg | Neg | 22.6 | 20.8 | Neg | 34.9 | 21.6 | Neg |
| **SEER_057a** | 26 | 68 | 500 | Neg | Neg | 22.6 | 20.6 | Neg | Neg | 21.4 | Neg |
| **SEER_057b** | 26 | 68 | 100 | Neg | Neg | 22.2 | 20.5 | Neg | Neg | 21.0 | Neg |
| **SEER_057c** | 26 | 68 | 25 | Neg | Neg | 22.8 | 20.5 | Neg | Neg | 21.3 | Neg |
| **SEER_058** | 12 | 51 | 500 | Neg | Neg | 22.9 | 20.7 | Neg | Neg | 21.5 | Neg |
| **SEER_059** | 12 | 53 | 500 | Neg | Neg | 23.1 | 20.9 | Neg | Neg | 21.6 | Neg |
| **SEER_061** | 50 | 86 | 500 | Neg | Neg | 22.6 | 20.9 | Neg | Neg | 21.6 | Neg |
| **SEER_063** | 27 | 77 | 500 | Neg | Neg | 22.2 | 20.5 | Neg | Neg | 21.1 | Neg |
| **SEER_064** | 42 | 73 | 500 | Neg | Neg | 23.6 | 21.0 | Neg | Neg | 23.8 | Neg |
| **SEER_065** | 21 | 64 | 500 | Neg | Neg | 22.0 | 20.5 | Neg | Neg | 21.1 | Neg |
| **SEER_066** | 12 | 56 | 500 | Neg | Neg | 22.1 | 20.5 | Neg | Neg | 21.1 | Neg |
| **SEER_067a** | 24 | 71 | 500 | Neg | Neg | 22.0 | 20.6 | Neg | Neg | 21.1 | Neg |
| **SEER_067b** | 24 | 71 | 100 | Neg | Neg | 22.4 | 20.6 | Neg | Neg | 21.0 | Neg |
| **SEER_067c** | 24 | 71 | 25 | Neg | Neg | 22.4 | 20.4 | Neg | Neg | 21.0 | Neg |
| **SEER_067d** | 24 | 71 | 10 | Neg | Neg | 24.0 | 20.7 | Neg | Neg | 21.1 | Neg |

**S2 Table footnotes:** **HK** = Housekeeping gene controls (ACTB, HPRT1); **RTC** = Reverse Transcription Control (tests for the presence of RT inhibitors using a built-in external RNA control); **PPC** = Positive PCR Control (plasmid template with primers for detection, tests for the presence of PCR inhibitors in the presence (cDNA) or absence (H_2_O) of experimental template); **GDC** = Genomic DNA Control (detects non-transcribed genomic DNA contamination); **NRT** = No Reverse Transcription (tests for genomic DNA contamination by amplification of a housekeeping gene directly from the RNA sample); **NTC** = No Template Control (tests for DNA contamination during experimental setup by trying to detect an abundant housekeeping gene). The a, b, c, and d for SEER 18, 57, and 67 for the qPCR represent reactions where the same input sample was used, but at different input amounts. This was done to provide replicates and also to check the lower limit where the assay would work for the SEER samples. SEER 18a, 57a, and 67a had 500 ng input like the rest of the samples. “b” samples for each of these were used at 100 ng input. The input amount amount 18c was also 100 ng, to provide a technical replicate for SEER 18b. SEER 57c, 67c and SEER 18d were used at 25 ng input. Optimum detection and amplification results in C_T_ values between 20 and 30, with the lower values indicating earlier detection, and therefore, higher expression levels. C_T_ values above 35 are marked as ‘Neg’ (negative).
