# Supplementary material for: Robustness of RNA sequencing on older formalin-fixed paraffin-embedded tissue from high-grade ovarian serous adenocarcinomas
Source: PLoS One. 2019 May 6;14(5):e0216050. doi: 10.1371/journal.pone.0216050 (PMC6502345; doi:10.1371/journal.pone.0216050)
Supplement: S3 Table — A: Conserved genes in samples across specimen storage times and SEER sites. B: KEGG Pathway of 189 Highly Expressed Genes. (DOCX) [file pone.0216050.s007.docx]

**Part A of S3 Table: Conserved genes in samples across specimen storage times and SEER sites.**

| ID | Gene Name | ID | Gene Name | ID | Gene Name |
| --- | --- | --- | --- | --- | --- |
| ENSG00000116133 | 24-dehydrocholesterol reductase(DHCR24) | ENSG00000167658 | eukaryotic translation elongation factor 2(EEF2) | ENSG00000154358 | obscurin, cytoskeletal calmodulin and titin-interacting RhoGEF(OBSCN) |
| ENSG00000154734 | ADAM metallopeptidase with thrombospondin type 1 motif 1(ADAMTS1) | ENSG00000114867 | eukaryotic translation initiation factor 4 gamma 1(EIF4G1) | ENSG00000125618 | paired box 8(PAX8) |
| ENSG00000185567 | AHNAK nucleoprotein 2(AHNAK2) | ENSG00000110321 | eukaryotic translation initiation factor 4 gamma 2(EIF4G2) | ENSG00000198300 | paternally expressed 3(PEG3) |
| ENSG00000124942 | AHNAK nucleoprotein(AHNAK) | ENSG00000063046 | eukaryotic translation initiation factor 4B(EIF4B) | ENSG00000100941 | pinin, desmosome associated protein(PNN) |
| ENSG00000058668 | ATPase plasma membrane Ca2+ transporting 4(ATP2B4) | ENSG00000180921 | family with sequence similarity 83 member H(FAM83H) | ENSG00000178209 | plectin(PLEC) |
| ENSG00000143384 | BCL2 family apoptosis regulator(MCL1) | ENSG00000087086 | ferritin light chain(FTL) | ENSG00000164050 | plexin B1(PLXNB1) |
| ENSG00000196776 | CD47 molecule(CD47) | ENSG00000115414 | fibronectin 1(FN1) | ENSG00000196576 | plexin B2(PLXNB2) |
| ENSG00000005339 | CREB binding protein(CREBBP) | ENSG00000136068 | filamin B(FLNB) | ENSG00000128567 | podocalyxin like(PODXL) |
| ENSG00000100201 | DEAD-box helicase 17(DDX17) | ENSG00000148180 | gelsolin(GSN) | ENSG00000173193 | poly(ADP-ribose) polymerase family member 14(PARP14) |
| ENSG00000173442 | EH domain binding protein 1 like 1(EHBP1L1) | ENSG00000135821 | glutamate-ammonia ligase(GLUL) | ENSG00000197111 | poly(rC) binding protein 2(PCBP2) |
| ENSG00000075426 | FOS like 2, AP-1 transcription factor subunit(FOSL2) | ENSG00000225151 | golgin A2 pseudogene 7(GOLGA2P7) | ENSG00000204469 | proline rich coiled-coil 2A(PRRC2A) |
| ENSG00000089280 | FUS RNA binding protein(FUS) | ENSG00000175265 | golgin A8 family member A(GOLGA8A) | ENSG00000130723 | proline rich coiled-coil 2B(PRRC2B) |
| ENSG00000170345 | Fos proto-oncogene, AP-1 transcription factor subunit(FOS) | ENSG00000173230 | golgin B1(GOLGB1) | ENSG00000117523 | proline rich coiled-coil 2C(PRRC2C) |
| ENSG00000125740 | FosB proto-oncogene, AP-1 transcription factor subunit(FOSB) | ENSG00000080824 | heat shock protein 90 alpha family class A member 1(HSP90AA1) | ENSG00000253729 | protein kinase, DNA-activated, catalytic polypeptide(PRKDC) |
| ENSG00000087460 | GNAS complex locus(GNAS) | ENSG00000096384 | heat shock protein 90 alpha family class B member 1(HSP90AB1) | ENSG00000142949 | protein tyrosine phosphatase, receptor type F(PTPRF) |
| ENSG00000086758 | HECT, UBA and WWE domain containing 1, E3 ubiquitin protein ligase(HUWE1) | ENSG00000142798 | heparan sulfate proteoglycan 2(HSPG2) | ENSG00000204628 | receptor for activated C kinase 1(RACK1) |
| ENSG00000269821 | KCNQ1 opposite strand/antisense transcript 1 (non-protein coding)(KCNQ1OT1) | ENSG00000135486 | heterogeneous nuclear ribonucleoprotein A1(HNRNPA1) | ENSG00000147403 | ribosomal protein L10(RPL10) |
| ENSG00000174718 | KIAA1551(KIAA1551) | ENSG00000122566 | heterogeneous nuclear ribonucleoprotein A2/B1(HNRNPA2B1) | ENSG00000197958 | ribosomal protein L12(RPL12) |
| ENSG00000123384 | LDL receptor related protein 1(LRP1) | ENSG00000169045 | heterogeneous nuclear ribonucleoprotein H1 (H)(HNRNPH1) | ENSG00000167526 | ribosomal protein L13(RPL13) |
| ENSG00000145012 | LIM domain containing preferred translocation partner in lipoma(LPP) | ENSG00000153187 | heterogeneous nuclear ribonucleoprotein U(HNRNPU) | ENSG00000142541 | ribosomal protein L13a(RPL13A) |
| ENSG00000085276 | MDS1 and EVI1 complex locus(MECOM) | ENSG00000137309 | high mobility group AT-hook 1(HMGA1) | ENSG00000174748 | ribosomal protein L15(RPL15) |
| ENSG00000275023 | MLLT6, PHD finger domain containing(MLLT6) | ENSG00000061273 | histone deacetylase 7(HDAC7) | ENSG00000063177 | ribosomal protein L18(RPL18) |
| ENSG00000143995 | Meis homeobox 1(MEIS1) | ENSG00000172534 | host cell factor C1(HCFC1) | ENSG00000100316 | ribosomal protein L3(RPL3) |
| ENSG00000147162 | O-linked N-acetylglucosamine (GlcNAc) transferase(OGT) | ENSG00000167244 | insulin like growth factor 2(IGF2) | ENSG00000071082 | ribosomal protein L31(RPL31) |
| ENSG00000189223 | PAX8 antisense RNA 1(PAX8-AS1) | ENSG00000115461 | insulin like growth factor binding protein 5(IGFBP5) | ENSG00000174444 | ribosomal protein L4(RPL4) |
| ENSG00000132424 | PNN interacting serine and arginine rich protein(PNISR) | ENSG00000132470 | integrin subunit beta 4(ITGB4) | ENSG00000229117 | ribosomal protein L41(RPL41) |
| ENSG00000202198 | RNA, 7SK small nuclear(RN7SK) | ENSG00000105855 | integrin subunit beta 8(ITGB8) | ENSG00000122406 | ribosomal protein L5(RPL5) |
| ENSG00000206652 | RNA, U1 small nuclear 1(RNU1-1) | ENSG00000129351 | interleukin enhancer binding factor 3(ILF3) | ENSG00000147604 | ribosomal protein L7(RPL7) |
| ENSG00000207005 | RNA, U1 small nuclear 2(RNU1-2) | ENSG00000203485 | inverted formin, FH2 and WH2 domain containing(INF2) | ENSG00000148303 | ribosomal protein L7a(RPL7A) |
| ENSG00000207513 | RNA, U1 small nuclear 3(RNU1-3) | ENSG00000135480 | keratin 7(KRT7) | ENSG00000163682 | ribosomal protein L9(RPL9) |
| ENSG00000207389 | RNA, U1 small nuclear 4(RNU1-4) | ENSG00000168056 | latent transforming growth factor beta binding protein 3(LTBP3) | ENSG00000142534 | ribosomal protein S11(RPS11) |
| ENSG00000202538 | RNA, U4 small nuclear 2(RNU4-2) | ENSG00000167615 | leukocyte receptor cluster member 8(LENG8) | ENSG00000105193 | ribosomal protein S16(RPS16) |
| ENSG00000206737 | RNA, variant U1 small nuclear 18(RNVU1-18) | ENSG00000160932 | lymphocyte antigen 6 complex, locus E(LY6E) | ENSG00000231500 | ribosomal protein S18(RPS18) |
| ENSG00000206585 | RNA, variant U1 small nuclear 7(RNVU1-7) | ENSG00000055609 | lysine methyltransferase 2C(KMT2C) | ENSG00000105372 | ribosomal protein S19(RPS19) |
| ENSG00000157106 | SMG1, nonsense mediated mRNA decay associated PI3K related kinase(SMG1) | ENSG00000167548 | lysine methyltransferase 2D(KMT2D) | ENSG00000149273 | ribosomal protein S3(RPS3) |
| ENSG00000282458 | WAS protein family homolog 5 pseudogene(WASH5P) | ENSG00000214548 | maternally expressed 3 (non-protein coding)(MEG3) | ENSG00000198034 | ribosomal protein S4, X-linked(RPS4X) |
| ENSG00000060237 | WNK lysine deficient protein kinase 1(WNK1) | ENSG00000251562 | metastasis associated lung adenocarcinoma transcript 1 (non-protein coding)(MALAT1) | ENSG00000137154 | ribosomal protein S6(RPS6) |
| ENSG00000229807 | X inactive specific transcript (non-protein coding)(XIST) | ENSG00000168906 | methionine adenosyltransferase 2A(MAT2A) | ENSG00000142937 | ribosomal protein S8(RPS8) |
| ENSG00000065978 | Y-box binding protein 1(YBX1) | ENSG00000249669 | microRNA 143(MIR143) | ENSG00000170889 | ribosomal protein S9(RPS9) |
| ENSG00000185650 | ZFP36 ring finger protein like 1(ZFP36L1) | ENSG00000062716 | microRNA 21(MIR21) | ENSG00000168028 | ribosomal protein SA(RPSA) |
| ENSG00000128016 | ZFP36 ring finger protein(ZFP36) | ENSG00000168542 | microRNA 3606(MIR3606) | ENSG00000137818 | ribosomal protein lateral stalk subunit P1(RPLP1) |
| ENSG00000149925 | aldolase, fructose-bisphosphate A(ALDOA) | ENSG00000204580 | microRNA 4640(MIR4640) | ENSG00000125844 | ribosome binding protein 1(RRBP1) |
| ENSG00000122359 | annexin A11(ANXA11) | ENSG00000130702 | microRNA 4758(MIR4758) | ENSG00000173821 | ring finger protein 213(RNF213) |
| ENSG00000182718 | annexin A2(ANXA2) | ENSG00000010244 | microRNA 632(MIR632) | ENSG00000113140 | secreted protein acidic and cysteine rich(SPARC) |
| ENSG00000123908 | argonaute 2, RISC catalytic component(AGO2) | ENSG00000159140 | microRNA 6501(MIR6501) | ENSG00000167642 | serine peptidase inhibitor, Kunitz type 2(SPINT2) |
| ENSG00000111676 | atrophin 1(ATN1) | ENSG00000120885 | microRNA 6843(MIR6843) | ENSG00000167978 | serine/arginine repetitive matrix 2(SRRM2) |
| ENSG00000187244 | basal cell adhesion molecule (Lutheran blood group)(BCAM) | ENSG00000161016 | microRNA 6850(MIR6850) | ENSG00000278249 | small Cajal body-specific RNA 2(SCARNA2) |
| ENSG00000122786 | caldesmon 1(CALD1) | ENSG00000070756 | microRNA 7705(MIR7705) | ENSG00000274266 | small nucleolar RNA, H/ACA box 73A(SNORA73A) |
| ENSG00000179218 | calreticulin(CALR) | ENSG00000180900 | microRNA 937(MIR937) | ENSG00000157765 | solute carrier family 34 member 2(SLC34A2) |
| ENSG00000117984 | cathepsin D(CTSD) | ENSG00000127603 | microtubule-actin crosslinking factor 1(MACF1) | ENSG00000115306 | spectrin beta, non-erythrocytic 1(SPTBN1) |
| ENSG00000108821 | collagen type I alpha 1 chain(COL1A1) | ENSG00000088888 | mitochondrial antiviral signaling protein(MAVS) | ENSG00000054654 | spectrin repeat containing nuclear envelope protein 2(SYNE2) |
| ENSG00000164692 | collagen type I alpha 2 chain(COL1A2) | ENSG00000181143 | mucin 16, cell surface associated(MUC16) | ENSG00000116560 | splicing factor proline and glutamine rich(SFPQ) |
| ENSG00000130635 | collagen type V alpha 1 chain(COL5A1) | ENSG00000145113 | mucin 4, cell surface associated(MUC4) | ENSG00000079308 | tensin 1(TNS1) |
| ENSG00000142156 | collagen type VI alpha 1 chain(COL6A1) | ENSG00000100345 | myosin heavy chain 9(MYH9) | ENSG00000136205 | tensin 3(TNS3) |
| ENSG00000182871 | collagen type XVIII alpha 1 chain(COL18A1) | ENSG00000114857 | natural killer cell triggering receptor(NKTR) | ENSG00000137801 | thrombospondin 1(THBS1) |
| ENSG00000125730 | complement C3(C3) | ENSG00000260032 | non-coding RNA activated by DNA damage(NORAD) | ENSG00000177565 | transducin beta like 1 X-linked receptor 1(TBL1XR1) |
| ENSG00000096696 | desmoplakin(DSP) | ENSG00000074181 | notch 3(NOTCH3) | ENSG00000182095 | trinucleotide repeat containing 18(TNRC18) |
| ENSG00000197102 | dynein cytoplasmic 1 heavy chain 1(DYNC1H1) | ENSG00000141905 | nuclear factor I C(NFIC) | ENSG00000140416 | tropomyosin 1 (alpha)(TPM1) |
| ENSG00000120738 | early growth response 1(EGR1) | ENSG00000137497 | nuclear mitotic apparatus protein 1(NUMA1) | ENSG00000167460 | tropomyosin 4(TPM4) |
| ENSG00000261150 | epiplakin 1(EPPK1) | ENSG00000245532 | nuclear paraspeckle assembly transcript 1 (non-protein coding)(NEAT1) | ENSG00000133112 | tumor protein, translationally-controlled 1(TPT1) |
| ENSG00000156508 | eukaryotic translation elongation factor 1 alpha 1(EEF1A1) | ENSG00000243716 | nuclear pore complex interacting protein family member B5(NPIPB5) | ENSG00000164924 | tyrosine 3-monooxygenase/tryptophan 5-monooxygenase activation protein zeta(YWHAZ) |
| ENSG00000104529 | eukaryotic translation elongation factor 1 delta(EEF1D) | ENSG00000196498 | nuclear receptor corepressor 2(NCOR2) | ENSG00000112715 | vascular endothelial growth factor A(VEGFA) |
| ENSG00000254772 | eukaryotic translation elongation factor 1 gamma(EEF1G) | ENSG00000123358 | nuclear receptor subfamily 4 group A member 1(NR4A1) | ENSG00000026025 | vimentin(VIM) |

**Part B of S3 Table: KEGG pathway analysis of the 189 highly expressed genes from S3 Table A**

| **Term** | **Count** | **%** | **P-Value** |
| --- | --- | --- | --- |
| **Ribosome** | **25** | **13.2** | **4.50E-20** |
| **PI3K-Akt signaling pathway** | **16** | **8.5** | **1.40E-04** |
| **ECM-receptor interaction** | **10** | **5.3** | **4.40E-06** |
| **Focal adhesion** | **10** | **5.3** | **3.10E-03** |
| **Proteoglycans in cancer** | **9** | **4.8** | **8.90E-03** |
| **Dilated cardiomyopathy** | **5** | **2.6** | **3.50E-02** |
| **Protein digestion and absorption** | **5** | **2.6** | **4.00E-02** |
| **MicroRNAs in cancer** | **9** | **4.8** | **5.80E-02** |
| **Amoebiasis** | **5** | **2.6** | **7.00E-02** |
