# Supplementary material for: Robustness of RNA sequencing on older formalin-fixed paraffin-embedded tissue from high-grade ovarian serous adenocarcinomas
Source: PLoS One. 2019 May 6;14(5):e0216050. doi: 10.1371/journal.pone.0216050 (PMC6502345; doi:10.1371/journal.pone.0216050)
Supplement: S4 Table — (DOCX) [file pone.0216050.s008.docx]

**S4 Table: Biological Replicates QC and Sequencing Statistics**

| **Sample** | **Replicates** | **RIN** | **DV100** | **Lib size- bp** | **Molarity- nM** | **Age of Samples** | **Total Reads** | **% Total Mapped Reads** | **% Non Duplicated Reads** | **Mapped Reads TPM > 1** | **Bacterial Mapped** | **%mRNA Bases** |
| --- | --- | --- | --- | --- | --- | --- | --- | --- | --- | --- | --- | --- |
| **SEER_045** | **SEER_045** | **2.4** | **55** | **368** | **4.6** | **9** | **21,915,749** | **61.43** | **3.03** | **7150** | **9.08** | **16.47** |
| **SEER_065** | **SEER_045_replicate** | **2.3** | **64** | **456** | **0.84** | **9** | **17,460,218** | **31.06** | **8.61** | **8673** | **23.83** | **5.41** |
| **SEER_057** | **SEER_057** | **2.2** | **68** | **571** | **1.4** | **21** | **4,753,788** | **33.79** | **15.49** | **9816** | **24.29** | **14.87** |
| **SEER_067** | **SEER_057_replicate** | **2.3** | **71** | **462** | **24** | **21** | **48,929,221** | **33.86** | **14.03** | **21102** | **40.39** | **10.73** |
| **SEER_058** | **SEER_058** | **2.4** | **51** | **479** | **1.3** | **21** | **3,991,454** | **33.11** | **13.73** | **7225** | **29.53** | **11.92** |
| **SEER_059** | **SEER_058_replicate** | **2.4** | **53** | **520** | **2** | **21** | **10,301,269** | **56.96** | **17.21** | **17563** | **18.60** | **5.48** |
| **SEER_050** | **SEER_050** | **2.4** | **59** | **376** | **6.6** | **14** | **28,768,619** | **46.91** | **5.02** | **7587** | **7.22** | **5.76** |
| **SEER_066** | **SEER_050_replicate** | **2.4** | **56** | **426** | **26** | **14** | **50,860,233** | **65.31** | **6.03** | **13356** | **21.89** | **11.75** |
| **SEER_005** | **SEER_005** | **1.1** |  | **382** | **1.53** | **24** | **42,470,014** | **53.38** | **2.19** | **11914** | **2.69** | **2.83** |
| **SEER_062** | **SEER_005_replicate** | **2.3** | **74** | **344** | **126** | **24** | **48,198,870** | **67.76** | **71.55** | **32501** | **0.41** | **21.74** |
| **SEER_007** | **SEER_007** | **1** |  | **408** | **1.43** | **18** | **38,999,981** | **57.96** | **1.96** | **2040** | **1.71** | **1.56** |
| **SEER_063** | **SEER_007_replicate** | **2.3** | **77** | **356** | **41** | **18** | **58,852,698** | **89.71** | **76.49** | **36465** | **0.49** | **17.43** |
| **SEER_020** | **SEER_020** | **2.3** | **75** | **413** | **10.3** | **7** | **159,424,876** | **51.72** | **2.33** | **21507** | **22.61** | **21.01** |
| **SEER_064** | **SEER_020_replicate** | **2.3** | **73** | **478** | **18.6** | **7** | **25,696,296** | **8.57** | **23.72** | **15029** | **55.47** | **13.51** |
